# An automated in vitro wound healing microscopy image analysis approach utilizing U-net-based deep learning methodology

**DOI:** 10.1186/s12880-024-01332-2

**Published:** 2024-06-25

**Authors:** Dilan Doğru, Gizem D. Özdemir, Mehmet A. Özdemir, Utku K. Ercan, Nermin Topaloğlu Avşar, Onan Güren

**Affiliations:** 1 Department of Biomedical Engineering, Graduate School of Natural and Applied Sciences, Izmir Katip Celebi University, Izmir, Turkey; 2 Department of Biomedical Engineering, Faculty of Engineering and Architecture, Izmir Katip Celebi University, Izmir, Turkey

**Keywords:** Wound healing, Microscopy, Segmentation, Deep learning, U-net, CNN

## Abstract

**Background:**

The assessment of in vitro wound healing images is critical for determining the efficacy of the therapy-of-interest that may influence the wound healing process. Existing methods suffer significant limitations, such as user dependency, time-consuming nature, and lack of sensitivity, thus paving the way for automated analysis approaches.

**Methods:**

Hereby, three structurally different variations of U-net architectures based on convolutional neural networks (CNN) were implemented for the segmentation of in vitro wound healing microscopy images. The developed models were fed using two independent datasets after applying a novel augmentation method aimed at the more sensitive analysis of edges after the preprocessing. Then, predicted masks were utilized for the accurate calculation of wound areas. Eventually, the therapy efficacy-indicator wound areas were thoroughly compared with current well-known tools such as ImageJ and TScratch.

**Results:**

The average dice similarity coefficient (DSC) scores were obtained as 0.958$\sim$0.968 for U-net-based deep learning models. The averaged absolute percentage errors (PE) of predicted wound areas to ground truth were 6.41%, 3.70%, and 3.73%, respectively for U-net, U-net++, and Attention U-net, while ImageJ and TScratch had considerable averaged error rates of 22.59% and 33.88%, respectively.

**Conclusions:**

Comparative analyses revealed that the developed models outperformed the conventional approaches in terms of analysis time and segmentation sensitivity. The developed models also hold great promise for the prediction of the in vitro wound area, regardless of the therapy-of-interest, cell line, magnification of the microscope, or other application-dependent parameters.

**Supplementary Information:**

The online version contains supplementary material available at 10.1186/s12880-024-01332-2.

## Background

The term “wound” often refers to the destruction of the epithelial tissue’s integrity brought on by any degree of violence, pressure, or other external factors [1, 2]. Wound healing is a dynamic process that might result in various infections and complications at each stage [3, 4]. These stages might be prolonged by any infection or necrosis that takes place during the process, leading to chronic wounds [5]. Depending on the severity of the infection, there is a risk of amputation in chronic wounds caused by diabetes [2]. Besides affecting people’s quality of life, wounds may lead to emotional, physical, and financial devastation [6]. Furthermore, additional expenses associated with chronic wounds, such as longer hospitalization and the need for specialized, expensive wound care products, raise medical costs [7]. Therefore, the treatment of wounds is essential. Traditional and novel therapies both have a critical role in wound healing, and various methods for wound treatment have been investigated comprehensively [8–12]. Despite the widespread usage of traditional medical therapies worldwide, novel therapies are becoming more crucial due to the benefits they provide. The novel therapies, although not limited, include light applications [8, 9], tissue engineering-based approaches [10], cold atmospheric plasma (CAP) applications [11], and nanoparticle-based treatment approaches [12]. In order to assess the efficacy of the methods used in these investigations, the wound-healing process must be monitored continuously [13]. Since the wound healing process in the human body involves a complex series of chemical and physical interactions happening simultaneously, the observation and monitoring of wounds have become challenging [14]. Therefore, as an initial step, the effectiveness of the treatment of interest must be evaluated by the examination of the data obtained from the in vitro wound model.

Numerous in vitro modeling techniques are used for different stages of wound healing, and the techniques can be modified to match the appropriate stage [15–17]. The scratch assay for 2D cell migration investigations [15], 3D wound healing assays [16], and Microphysiological Systems (MPS) [17] based on reconstructing a specific structure beneath 3D assays, are among the methodologies frequently used for wound healing migration stage modeling that is the focus of this study. Although 3D wound healing assays are more effective for obtaining complex migration and signaling structures in the in vivo microenvironment, they require expensive materials, scaffold fabrication, and a longer time to complete the process due to the complicated procedures [18]. Therefore, a variety of state-of-the-art approaches for modeling wound healing benefit from the 2D scratch assay method, which offers basic components and ease of application [15]. The scratch assay procedure, which is also known as the cell migration assay or wound healing assay, requires simple cell culture applications to construct 2D cell layers for tissue modeling [19]. The wound model is developed by scratching the confluent cells with a mechanical effect (usually through a pipette tip) and disrupting their integrity [15]. The cells are exposed to therapy-of-interest, and the cells’ migration is monitored at different time intervals. The cells migrate to the wound area over time, and thus the decrease in the wound area is used to evaluate the success of the treatment [20]. Therefore, the analysis of the images obtained during the in vitro wound healing monitoring is of great importance.

The conventional approaches rely on applying image processing techniques to in vitro wound healing images that are obtained at particular time intervals. These techniques depend on plots along the wound edges and computing the distance to determine the boundaries of the wound area [21]. However, one major drawback of manual calculations is that they often consider uneven edges as straight lines, which does not provide accurate results. Moreover, the middle portion of the wound gets smaller as it heals, and uneven progressions on the wound borders might happen. This may lead to an even more inaccurate determination of wound boundaries and, thus, wound area. Furthermore, the number of images increases over time due to the images being captured consecutively to monitor the migration and determine the effectiveness of treatment. Given that the analyses are performed manually, this process is time-consuming and more prone to errors. To lessen user-induced error in manual approaches and to automate the analysis, a variety of applications, such as ImageJ, Tscratch, MultiCellSeg, and MATLAB^®^ toolboxes, have been developed.

One of the most popular platforms for image processing applications is MATLAB^®^. Two of MATLAB’s toolboxes that are frequently used in image segmentation are the Texture Segmentation Algorithm [22] and Image Processing [23]. In the texture segmentation algorithm, the density difference in 11x11 pixel frames is determined for each pixel and analyzed with the texture filter algorithm. High pixel densities are determined as cellular regions and low pixel densities are determined as cell-free regions [24]. Similar principles are valid for the image processing toolbox. However, the map produced by computing the standard deviation of pixels in a moving window is used to determine the locations of cells and cell-free regions by applying a threshold [23]. Here, two windows-one large and one small-must be applied, and the intersection of the values calculated from these windows determines the wound area. With this, it is intended to reduce the noise and make the wound borders smoother. Automatic Invasiveness Measure (AIM), developed by Cortesi et al. [20], is a MATLAB-based tool that uses the local entropy difference between cell and cell-free regions. In this approach, the analysis was carried out with formulas based on gray-level probabilities by converting information from neighboring pixels of the current pixel into numerical parameters. The efficiency of their model was compared with that of the expert’s manual calculations which consisted of approximating the wound area with a rectangle shape via ImageJ, and TScratch programs. They asserted that their tool aligns with TScratch in terms of wound region identification and outperforms both their manual method and ImageJ in analysis speed, but is slightly behind TScratch [20].

Previous studies in the literature and current applications have utilized various image features to segment in vitro wound healing images [20, 23–25]. The majority of these approaches base their conclusions on assumptions about cell behavior, such as migration rate, movement direction, and cell density, which are determined through image processing operations. However, these methods often require many user-adjustable parameters, which can lead to variations in results and require additional time to determine optimal settings. As an indication, the majority of those tools lack cell separation [26], and when cells are marked, regardless of their morphology, all cells are assumed to have a circular shape. Cells naturally exhibit spindle movements during migration [27, 28], where their shape appears branched due to this spindle formation. These cells were also converted into circular shapes, and cells with radii that were lower than the radius value specified by the user were not accounted as cells. Recently, artificial intelligence (AI)-based approaches have been developed to address these limitations. These approaches can be divided into two categories: machine learning (ML) and deep learning (DL). While these AI-based methods have the potential to improve accuracy and efficiency, they also have their own limitations, such as poor prediction accuracy and effectiveness under certain conditions. For instance, in ML applications, inaccurate feature selection during feature extraction or improper feature map extraction may lead to low accuracy. On the other hand, for DL applications, problems such as low accuracy and high complexity are frequently reported [26, 29, 30], which may result from the distribution of the dataset used in the training phase or limited observations and may cause poor generalizability of the model in testing with new unseen data [31]. These shortcomings, which are present in both conventional and AI-based approaches referenced in the existing studies, might have a negative impact on the outcome of the research that examines the effects of applied therapy on the wound healing process. Therefore, there is still room for a standardized, generalizable, and automated fast analysis approach that is not affected by the applications in the images to be analyzed, the adjustments used in the applications, or the user’s preferences. Based on these, the following goals were established for each stage of the wound healing investigations in the scope of this study:

1. To monitor wound healing regardless of the cell line, therapy-of-interest, wound or scratch area, and magnification of the microscope,
2. To be able to associate therapy-of-interest effectiveness on wound healing with certain quantitative parameters where the concept of the specific therapy dose is not entirely/sensitively specified.

Considering these goals, a novel DL-based approach has been developed to segment the wound area accurately, automatically, and with as minimal human involvement as possible. More complex U-net++ and Attention U-net structures were also employed in addition to the developed model, using the 5-layer U-net structure, and it has been extensively investigated whether alterations in these deep structures affect the results and also comprehensively compared with image processing-based tools, ImageJ and TScratch.

## Materials and methods

**Fig. 1:**
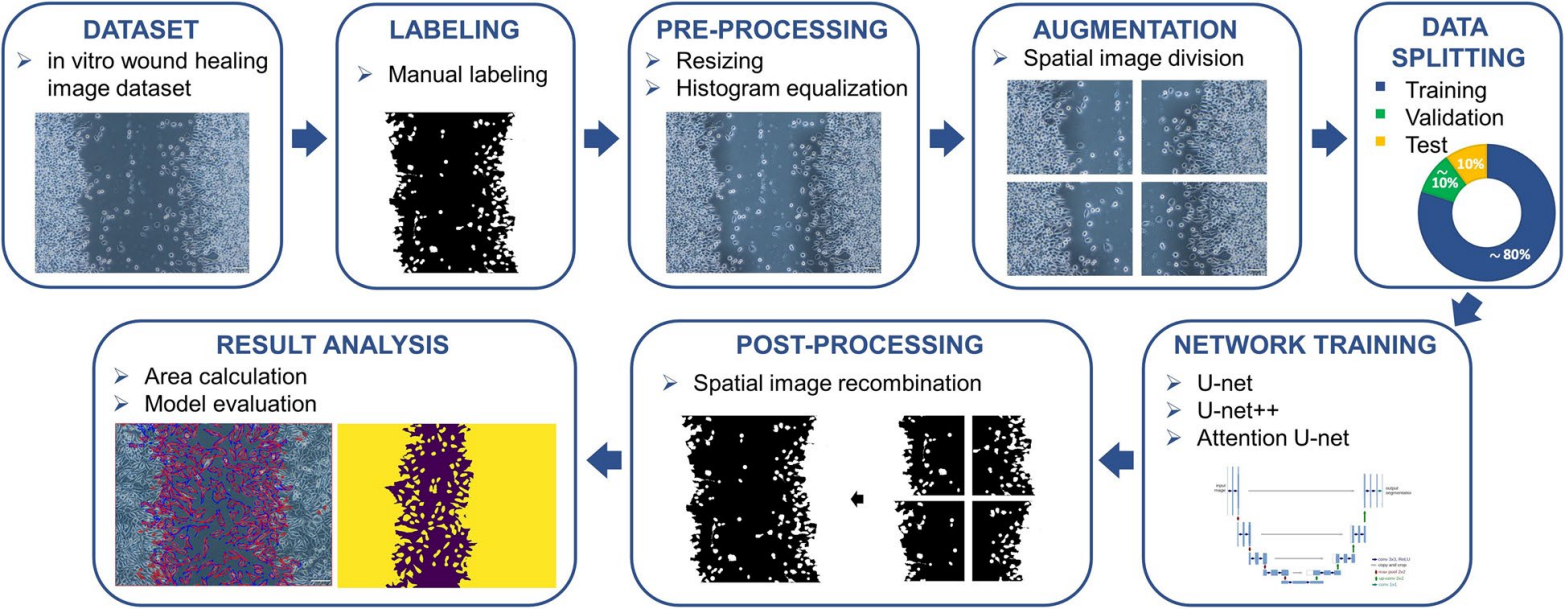
The workflow of the study shows the main steps taken to develop and evaluate the DL models for in vitro wound healing image segmentation

The visual workflow of the proposed methodological structure is presented in Fig. 1. A detailed description of each stage is given in the following.

### Datasets

In this study, a total of 400 images with 2556x1910 pixels from low-level laser therapy (LLLT) and CAP treatment in vitro wound healing investigations were used to construct the model. Out of these 400 images, 233 represent LLLT-treated healthy mouse fibroblast cell line (L929) cell line images at 40x and 100x magnification, and 167 represent CAP-treated cell lines. In vitro wound healing images from LLLT investigations conducted by the Izmir Katip Celebi University (IKCU) Biomedical Optics and Laser Applications Laboratory and in vitro wound healing images from CAP treatment obtained from the IKCU Plasma Medicine Laboratory were utilized. Brief explanations of these methodologies are given in Additional file 1. For the LLLT, images were obtained by using the scratch assay procedure on the L929 cell line irradiated by 655 nm and 808 nm diode lasers three times, each at an interval of 24 hours. In vitro wound healing images were obtained by capturing the healing process at 12-hour intervals at 40x and 100x magnifications with an inverted microscope (Olympus CKX41). A total of 233 RGB images with a size of 2556x1910 pixels were obtained at 6 different intervals between 0^th^ and 60^th^ hours were used in this study. The in vitro wound healing images from CAP treatment represent two different cell lines to evaluate the migration efficacy of CAP treatment and compare it with healthy and cancer cell lines. The human keratinocyte (HS2) cell line and squamous cell carcinoma (SCC) cell lines were treated with CAP operated at peak-to-peak voltage and 1 kHz frequency at different treatment times. In vitro wound healing images were obtained at 24-hour intervals at 40x and 100x magnifications using the same inverted microscope (Olympus CKX41) [32]. Among the 167 CAP-treated images, 133 were of the HS2 cell line, of which 67 were captured using a 40x magnification and 66 using a 100x magnification setting. The remaining 34 images were of the SCC cell line, with 15 captured at a 40x magnification and 19 at a 100x magnification setting. The details of the collected data are provided in Table 1. It can be observed that the dataset is composed of 400 images, of which 58.25% are from LLLT and 41.75% are from CAP treatment. Within the CAP treatment images, approximately 79% are microscopy images of the HS2 cell line at 40x and 100x magnifications, while the remaining 21% are the SCC cell line at the same magnifications.

**Table 1:** Descriptions of acquired data

Dataset	Cell line	Magnification	Acquired images	Augmented images
LLLT	L929	100x	68	272
		40x	165	660
CAP	HS2	100x	66	264
		40x	67	268
	SCC	100x	19	76
		40x	15	60
**Total**			400	1600

### Labeling

An expert created handcrafted annotations using a manual labeling tool, MATLAB^®^ R2021b Image Segmenter Toolbox, without utilizing any automated or parameter-based applications. A total of 400 RGB images were labeled by meticulously marking each point of the wound edges and cell lines, with an average processing time of 45 min per image. Rather than using the conventional method of drawing a vertical line from the leading edge, the labeling procedure identified the migrated cells to the wound region based on the Region of Interest (ROI). The borders of cells were meticulously identified in accordance with the orientation of the cell and were considered ROIs. As a result, the 400 binary masks that were utilized as ground truth during the model training and had the same dimensions as the 400 original images were obtained. In these binary masks, cell-containing areas, or ROIs, were indicated as white, while areas that were considered wound regions were indicated as black colors (Fig. 1).

### Pre-processing

A custom Python 3.7 script based on the scikit-image 0.18.3 module was used to apply the Contrast Limited Adaptive Histogram Equalization (CLAHE) [33] to the 400 RGB images in the datasets. This method was utilized not only to reduce the tonal variations caused by the image-capturing process but also to mitigate the shading effect often observed in the microscopy images (Fig. 1). As reported by Qiu et al. [34], CLAHE is the most suitable and effective image processing parameter for automatically determining the required contrast for an image. The CLAHE operation clips the pixel frequencies with a predefined clip limit, which is set according to the visibility of the image [35]. In this study, a clip limit of 0.005 was set to equalize the histogram of 400 RGB images. The histogram-equalized images were scaled down to 1024x768 pixels using the $INTER\_AREA$ interpolation function from OpenCV library [36]. This method employs pixel-area relations for resampling and is considered the best method for downscaling, as it utilizes an area-based (or pixel-area relation) image scaling algorithm [36]. It calculates the pixel value in the downscaled image as a weighted average of pixels in the nearest 2x2 neighborhood of the original image. The weights are determined by the areas of intersection between the original pixel and the pixel in the downscaled image. This approach ensures that the total brightness of the image is preserved and minimizes aliasing artifacts [36].

### Data augmentation

Contemporary studies in the literature indicate that utilizing a larger and more diverse dataset by expanding the dataset has a significant effect on the model, resulting in more accurate outcomes [37, 38]. To address the issue of heterogeneity in biomedical image datasets and improve the performance of the trained DL model, various augmentation strategies such as geometric transformations, scaling, and generating synthetic data using Generative Adversarial Networks (GANs) [39–42] were employed previously. In light of this, the strategy of dividing larger images into smaller parts [43], a similar method proposed by Serre et al. [44], has been adopted in this study. In this context, the preprocessed images along with their corresponding masks were spatially divided into four equal parts, ensuring that each part contained regions with both the wound and cells. This division process aimed to increase the number of training data and more efficiently/sensitively detect the edges between cells, resulting in a total of 1600 images. This strategy also helps mitigate the risk of overfitting and poor prediction outcomes by increasing the number of images available for model training [45].

### Data splitting

10% of the preprocessed images (40 out of 400) were randomly selected as a test set. The remaining 360 preprocessed images were randomly divided into training and validation sets at a 90:10 ratio. Consequently, approximately 90% of the data (325 images) were used for training, while 10% (35 images) were reserved for validation. The 40 images that were randomly selected for testing were grouped as follows: 25 L929 cell line images under 100x magnification were obtained from LLLT; 5 HS2 cell line images under 100x magnification and 5 HS2 cell line images under 40x magnification were obtained from CAP treatment; 3 SCC cell line images under 100x magnification and 2 SCC cell line images under 40x magnification were obtained from CAP treatment. For the validation images, these groups were determined randomly as 20 L929 cell line images under 100x magnification obtained from LLLT; 5 HS2 cell line images under 100x magnification and 5 HS2 cell line images under 40x magnification obtained from CAP treatment; 3 SCC cell line images under 100x magnification and 2 SCC cell line images under 40x magnification obtained from CAP treatment. After spatial splitting, a total of 1440 512x384 RGB images and their corresponding masks of the same size were used for the training phase. The same procedure was implemented for the images determined for testing and validation, resulting in a total of 160 images for testing and 140 images for validation. Before training, all images in digital form were converted to the *float32* format representing each pixel as a 32-bit floating-point number which offers a broader range of values and higher precision compared to the *uint8* [46].

### Deep learning architectures

The segmentation of biomedical images might be challenging because of constraints such as the inability to accurately identify the cell boundaries in the images and the variances brought on by the generally non-standard instruments. The U-net model proposed by Ronneberger et al. [47] in 2015 has had a significant impact on the sensitive and accurate segmentation of biomedical images. However, due to the diversity of biomedical images and the variety of obstacles encountered, such as the inherent variability in biological structures, the presence of noise in the images, class imbalance issues, and the challenges posed by the high dimensionality of the data, models were required to be developed over time, and studies were conducted to improve the reliability of the findings. Based on the developed models, we adopted various U-net structures including the plain U-net model for assuming baseline. In this model, the input image is downsampled and converted to a compressed representation in the encoder structure of the U-net, which is followed by a sequence of upsampling layers. Upsampling layers decompress the learned feature map to the same dimensions as the original image. It also receives contextual information from the downsampling layers via skip connections. The semantic value of each pixel is then calculated by combining local and global image information [48]. Furthermore, structurally variated versions of U-net models such as U-net++ and Attention U-net were also utilized without modifying model parameters to investigate the effects of variations in the model structures on the outcome. Zhou et al. [49] introduced U-net++ in 2018 to obtain more accurate outcomes in medical image segmentation. Unlike U-net, this version allows the integration of maps at various scales and offers higher detail retention by enhancing the feature maps from the encoder and decoder before combining them [50]. The developers stated that U-net++ is separated from U-net in three main ways. First, the model bridges the semantic gap between the encoder and decoder with convolution layers in skip paths. Secondly, it has dense skip connections that improve gradient flow. Lastly, the model has a deep control structure that improves pruning. On the other hand, Oktay et al. [51] developed the Attention U-net by integrating an attention gate (AG) before combining the corresponding features in the encoder and decoder structure and adjusting the encoder’s output properties. By focusing on prominent features transmitted through the skip connection, this structure makes it easier to generate the gate signal to eliminate the response of unnecessary and noisy ambiguity in the connection [52]. The number of parameters and layers used for the plain U-net model is also valid for the training for U-net++ and Attention U-net so that only the effect of model structure can be fairly examined. The dense skip blocks in the U-net++ structure reduce the number of parameters significantly, while the attention module added to the U-net skip connections increases the number of parameters in the Attention U-net. Compared with the plain U-net, while this decreases the computational cost of the U-net++, it unlikely increases the computational cost of the Attention U-net.

We utilized the three different variations of U-net mentioned above along with various combinations of datasets to develop a well-structured model capable of accurately predicting wound areas. This approach also allowed us to assess the generalizability of the presented methodology. However, the outcomes of the trained models need to be a post-process step to the predicted mask because all models were fed with spatially divided image datasets, which were utilized to increase the precision of U-net models and based on more sensitive detection of edges. The four image patches that underwent the training and prediction stages therefore spatially merge for subsequent analysis using a similar approach in the augmentation phase upon completing the model training.

### Manual wound area calculation

To evaluate the results of the proposed automated method, additional manual analysis was conducted by using the most frequently used tools, ImageJ and TScratch, on the same validation and test sets. The wound area may be determined approximately and manually by the user via ImageJ/Fiji, an open-source Java program [26]. This tool has been extended with the white wave model. Thus, the model visualizes cell migration by utilizing variations in images that are collected at various time intervals. Using the absolute values of the pixel differences between the images, the migratory or stationary cells can be established [53]. ImageJ has several built-in functions that can be used for wound healing analysis. An expert calculated wound areas by using ImageJ as follows: The first step is converting images to 8-bit and applying a bandpass filter using a fast Fourier transform. Afterward, the image can be thresholded and filtered using a minimum filter with a radius value of 7 pixels. This process allows for the selection of the wound area manually and the measurement of the wound size as a pixel value.

On the other hand, the TScratch tool examines images of wound healing using a curve-based approach. It performs fast discrete curve transforms, utilizing statistical and graphical outputs to distinguish between cell and cell-free regions. Thus, the application incorporates information from the original image into the coefficient curve encoding [54]. TScratch also includes several additional features, such as the ability to track individual cells and measure cell migration rates. Unlike ImageJ, TScratch was specifically designed for wound healing analysis and offers a more streamlined process. After loading the image into the software, the user can define the initial wound area by drawing a line along the edge of the scratch. Next, the software automatically detects the remaining scratch area and calculates the percentage of the original wound that has been closed. The user can then adjust the threshold and fine-tune the analysis as needed.

### Experimental setup

Each DL model was trained from scratch, validated, and tested utilizing the datasets obtained for this study. In order to mitigate the potential imbalance caused by the data distribution (i.e., cell line or microscope magnification factor), the dataset was split into two sub-datasets. The models were independently trained on each sub-dataset and tested with the other, e.g., one trained with only LLLT images and tested with CAP data, and the other trained with only CAP treatment images and then tested on the LLLT dataset. This approach was designed to assess the model’s ability to generalize from one data source to another. This resulted in three independent observations: one using the LLLT images, one using the CAP treatment images, and one using a mixed dataset that combines the two subsets. To ensure a fair comparison among models using the mixed dataset, the validation indices from each fold in the 5-fold cross-validation were stored, and these indices were then utilized for each model’s validation. For each dataset, networks were constructed using 5-layer U-net, U-net++, and Attention U-net models with an input image size of 512x384x3. The models were developed via Python 3.7-based custom scripts with the Keras 2.8.0 and Tensorflow 2.8.2 libraries at the backhand. Each layer consisted of 2D convolution, batch normalization, and ReLU activation. In contrast to these layers, the output activation was achieved using the sigmoid function. The output was then transferred to the following layer after the dimensions were reduced in half through the use of a 2x2 max-pooling operation. Additional file 2 provides a detailed summary of the built model. The models were trained using a 10^-3^ learning rate with an exponential decay rate of 0.9 and Adam Optimizer with a batch size of 4 and an epoch number of 50. The dice loss function was used during the training process, and the training progress was monitored based on the dice value. The output probability maps of U-Net-based architectures were transformed into binary values to generate masks. A threshold value for binarization was set to 0.5, with values greater than this being considered as 1. All these computations were performed on a computer equipped with an NVIDIA GeForce RTX 3080 Ti with a 12 GB GPU and 64 GB of RAM. In order to fairly isolate the effect of the model structure on the segmentation performances, the common network and training parameters (i.e., kernel sizes, epochs, batch sizes, etc.) were set to the same values, other network parameters (e.g., attention activations and deep supervisions) were set to their defaults, and operational system conditions were kept as constant as possible.

### Performance evaluation

The DSC, accuracy (ACC), intersection over union (IoU), precision (PRE), recall (REC), Receiver Operating Characteristics-Area Under The Curve (ROC-AUC), and specificity (SPE) metrics [45] were calculated to evaluate the model’s performance. Computation methods for these metrics are given as follows.

1 $$\begin{aligned} DSC&=\frac{2\cdot TP}{FP+2\cdot TP+FN}\end{aligned}$$

2 $$\begin{aligned} ACC&= \frac{TP+TN}{TP+TN+FP+FN}\end{aligned}$$

3 $$\begin{aligned} IoU&=\frac{TP}{TP+FP+FN}\end{aligned}$$

4 $$\begin{aligned} PRE&=\frac{TP}{TP+FP}\end{aligned}$$

5 $$\begin{aligned} REC&=\frac{TP}{TP+FN}\end{aligned}$$

6 $$\begin{aligned} SPE&=\frac{TN}{TN+FP} \end{aligned}$$

where *TP*, *TN*, *FP*, and *FN* indicate predicted classes as True Positive (image pixels that are correctly predicted as wound area), True Negative (image pixels correctly predicted as cells), False Positive (image pixels falsely predicted as wound area), and False Negative (image pixels falsely predicted as cells), respectively. Utilizing the pixels’ intensity in the microscopy images, the area of each predicted and ground truth binary label was calculated. The cell region was determined using a pixel intensity equal to 1, and the cell-free region (wound area) was determined using a pixel intensity equal to 0. The total wound area was calculated by using the following formula:

7 $$\begin{aligned} WA = \left\{ \begin{array}{ll} \sum \limits _{m,n=1}^{M,N} P_{(m,n)},&\textrm{if}\, P_{(m,n)} = 0 \end{array}\right. \end{aligned}$$

where *P* indicates the binary image, *M,N* is the image sizes, $P_{(m,n)}$ denotes the pixel intensity, and *WA* is the wound area. In order to compare wound area calculation performance, the percentage error (*PE*) between predicted areas and ground truth was calculated as follows:

8 $$\begin{aligned} \%\, error\, (PE) = \frac{WA_{(P)}-WA_{(GT)}}{WA_{(GT)}} \,x\, 100 \end{aligned}$$

where $WA_{(P)}$ indicates the predicted wound area and $WA_{(GT)}$ denotes the ground truth area. It should also be noted that *PE* values were calculated without their absolute because to observe the directions.

## Results

### Internal validation and testing

U-net, Unet++, and Attention U-net models were trained for each dataset. The normalized elapsed times, which are calculated based on the lowest value of the training, validation, and test phases, as well as the wound area analysis time, are presented in Table 2. Upon analyzing the normalized times for training with both datasets in Table 2, it is noticeable that the training time of U-net++ is approximately 12% faster than that of U-net, while Attention U-net is nearly equivalent to U-net, being just 1% faster. In the comparison of testing time, U-net and Attention U-net took about 10% and 5% longer, respectively, than the U-net++ model. Regarding the calculation time for testing, U-net++ and Attention U-net are both approximately 7% slower than U-net. For the validation time, Attention U-net is around 6% slower than U-net, while U-net++ is nearly equivalent to U-net. Finally, in terms of the calculation time for validation, both U-net++ and Attention U-net exhibited a minor reduction compared to U-net, being roughly 2% slower.

**Table 2:** Elapsed times in train, validation, and test phases, and wound area analysis times according to proposed models (the time intervals were normalized using the smallest value in each corresponding phase)

Metric	U-net	U-net++	Attention U-net
Train time	1.12	1.00	1.11
Test time	1.10	1.00	1.05
Calculation time (test)	1.00	1.07	1.07
Validation time	1.00	1.01	1.06
Calculation time (val.)	1.00	1.02	1.02

**Fig. 2:**
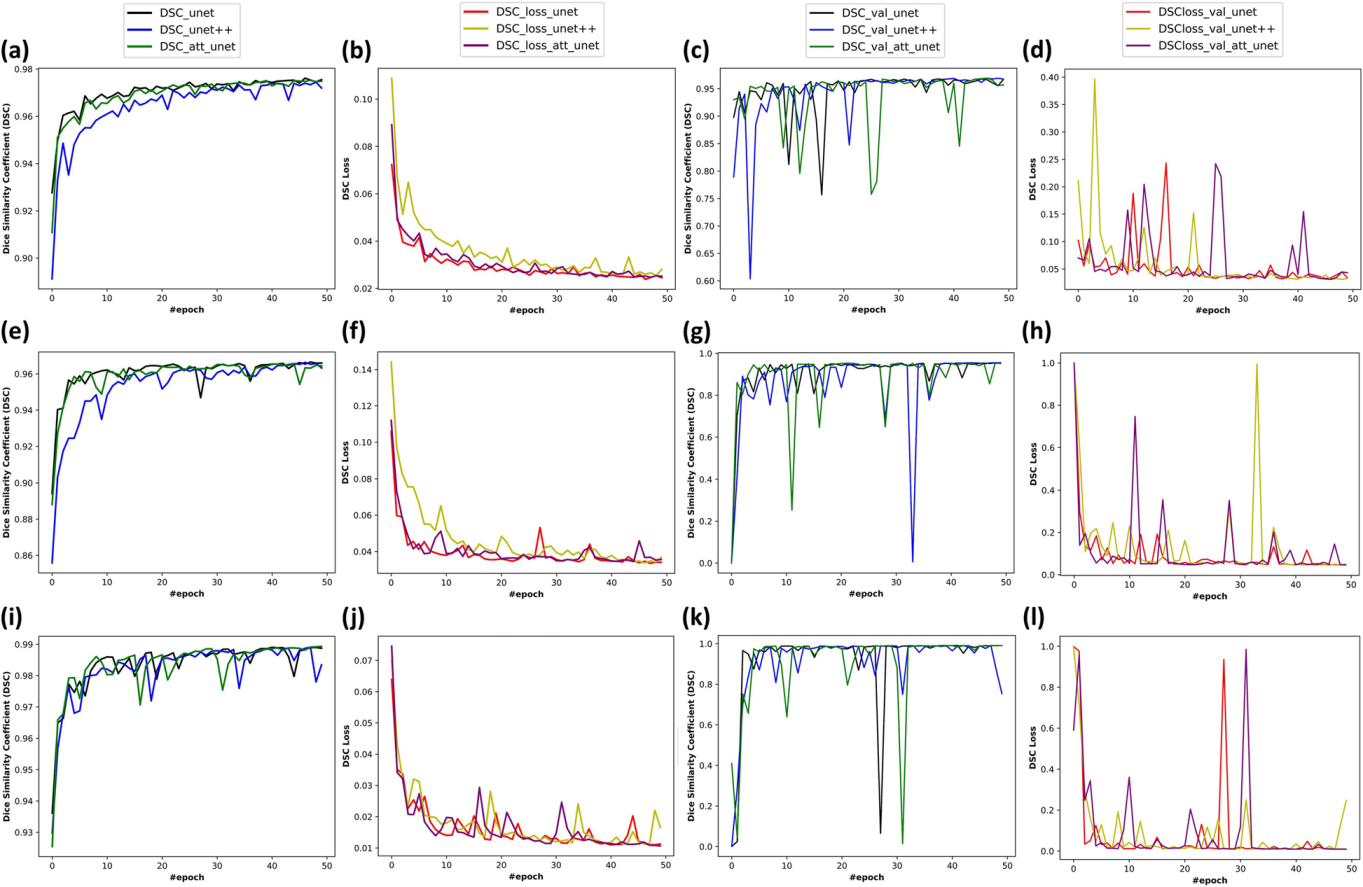
DSC and DSC-loss curves during the training and validation phases of different datasets: **a** DSC curves during training of combined dataset, **b** DSC-loss curves during training of combined dataset; **c** DSC curves for the validation of combined dataset, **d** DSC-loss curves for the validation of combined dataset; **e** DSC curves during training of the LLLT dataset, **f** DSC-loss curves during training of the LLLT dataset; **g** DSC curves for the validation of the LLLT dataset, **h** DSC-loss curves for the validation of the LLLT dataset; **i** DSC curves during training of the CAP dataset, **j** DSC-loss curves during training of the CAP dataset; **k** DSC curves for the validation of the CAP dataset, **l** DSC-loss curve for the validation of the CAP dataset

The models estimated spatially divided test and validation images, which underwent the same preprocessing as the training images, and then reconstructed them to provide prediction masks. Figure 2 presents the training results of the 5-layer U-net++ model and the DSC and loss values for each model across the 50 training epochs. The same figure shows the DSC and loss values across epochs for three different datasets. The detailed outcomes for the sub-datasets are shown in Table 3. Each used dataset produced approximate performance metrics results across the three models. Although the CAP dataset size was smaller than the LLLT dataset, the DSC results obtained from the CAP dataset outperformed the LLLT dataset by approximately 4% for validation and 5% for test sets. The ACC score of approximately 2% for validation produced the lowest difference between the metrics and the IoU score of about 6% produced the highest difference with the superiority of the CAP dataset. Similar results were observed in the test metrics. On the other hand, there is no significant difference between all three model performances across datasets (one-way ANOVA [37]; *p* $\ge$ .351). The DSC value, as a result of the test data, was 0.939 for both U-net and U-net++, and 0.941 for the Attention U-net model when using the LLLT data. Meanwhile, the DSC value for the test using CAP data was recorded as 0.991 for both U-net and U-net++, and 0.992 for the Attention U-net model. In the training phase with CAP data, it was noted that the U-net++ model finished 15% faster than the conventional U-net model and 14% faster than the Attention U-net model. This model required 38% more time to train with LLLT images compared to the CAP dataset. This gap widened to 59% for the conventional U-net and 56% for the Attention U-net in models trained with LLLT images. Upon internal evaluation, it was found that the discrepancy between the models trained with the LLLT dataset was 21% with the conventional U-net and 14% with the Attention U-net. The U-net++ model was observed to complete the validation and testing phases in less time compared to other models. However, the validation phase of the Attention U-net model took 3% more time in the CAP dataset and 5% more time in the testing phase than the conventional U-net model. This led to an extra 8% time for the Attention U-net in the LLLT dataset validation phase and a 5% in the testing phase. The calculation time, which signifies the duration from estimating a single image to calculating its area, was shorter for U-net++ in the CAP dataset and for the conventional U-net model in the LLLT dataset. The evaluation metric results and the calculation time for the wound area of the images were also included in Table 3. As a result, CAP treatment dataset results achieved superior performance compared to the LLLT dataset for all U-net-based models, but the difference was not significant.

**Table 3:** Performance score comparison of LLLT and CAP treatment datasets according to test and validation phases for proposed models (time intervals were presented as s)

		LLLT dataset			CAP treatment dataset		
Phase	Metric	U-net	U-net++	Attention U-net	U-net	U-net++	Attention U-net
Validation	DSC	0.953	0.954	0.953	0.991	0.991	0.991
	ACC	0.966	0.968	0.967	0.987	0.987	0.987
	IoU	0.914	0.919	0.916	0.981	0.982	0.982
	PRE	0.971	0.963	0.962	0.994	0.993	0.993
	REC	0.936	0.949	0.947	0.987	0.989	0.989
	ROC-AUC	0.927	0.924	0.921	0.974	0.974	0.974
	SPE	0.941	0.923	0.921	0.962	0.958	0.960
	Train time	1.59	1.38	1.56	1.15	1.00	1.14
	Validation time	1.17	1.15	1.25	1.03	1.00	1.06
	Calculation time	2.06	3.23	3.24	1.97	1.00	1.08
Test	DSC	0.939	0.939	0.941	0.991	0.991	0.992
	ACC	0.946	0.947	0.949	0.987	0.987	0.988
	IoU	0.893	0.895	0.900	0.982	0.982	0.984
	PRE	0.966	0.954	0.953	0.995	0.994	0.994
	REC	0.918	0.930	0.937	0.987	0.988	0.990
	ROC-AUC	0.927	0.919	0.920	0.958	0.952	0.960
	SPE	0.936	0.907	0.902	0.929	0.915	0.931
	Test time	1.41	1.34	1.46	1.04	1.00	1.09
	Calculation time	1.12	4.00	4.02	1.66	1.00	1.04

**Fig. 3:**
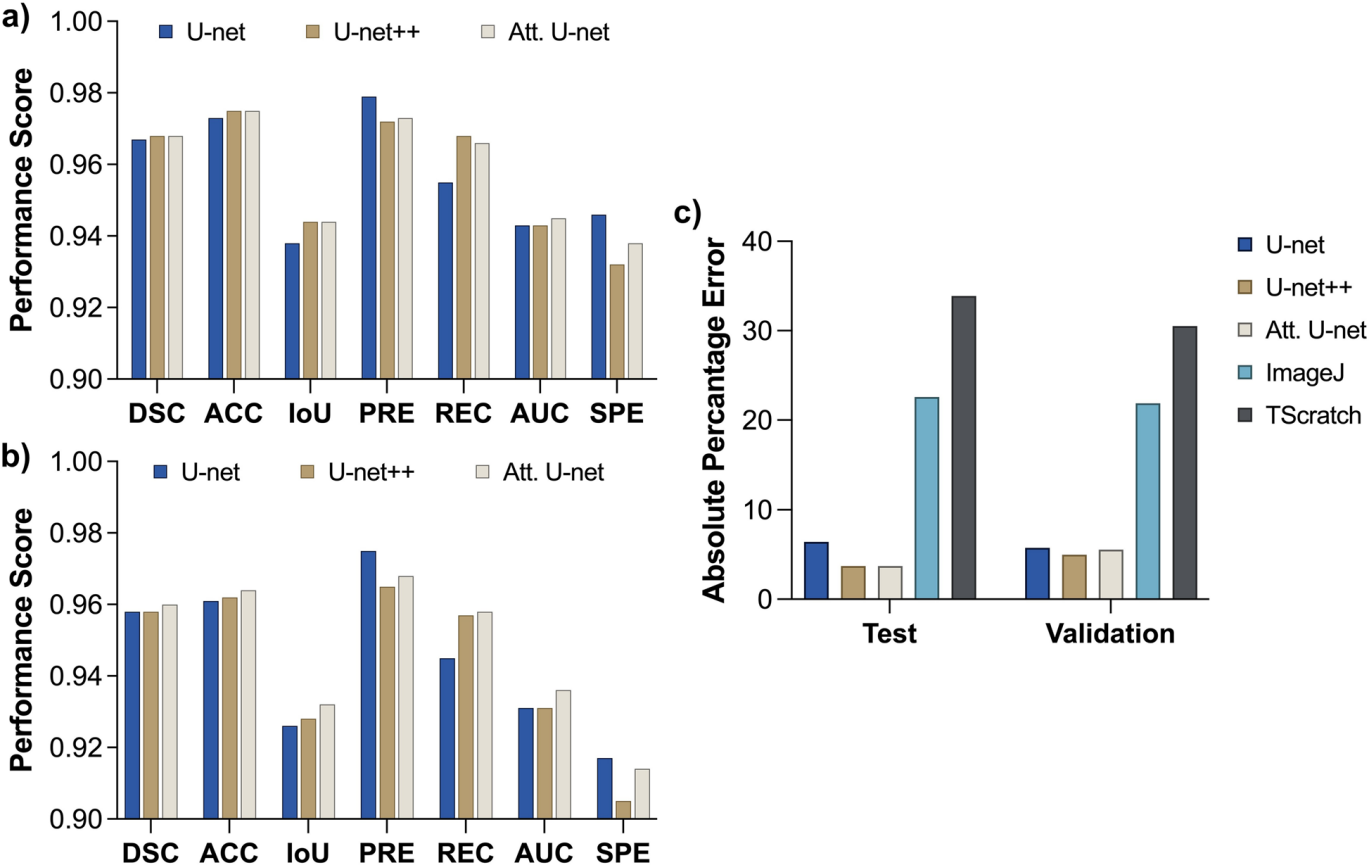
Performance evaluation of the developed models based on **a** validation scores, **b** test scores, and **c** absolute PEs in comparison with currently available tools

Figure 3a and b present the model performance metrics obtained from the prediction images of mixed datasets. The overall ACC value was calculated as 0.961, and the mean DSC value of the 40 images was calculated as 0.958 for the U-net and U-net++, and 0.960 for the Attention U-net, respectively. U-net++ was 1% behind the U-net model for the PRE score in test data, whereas the Attention-based model had 0.3% lower PRE. In regard to validation scores, these rates were determined to be 0.7% and 0.1% behind U-net and Attention U-net, respectively. All models had approximately 2%-8% lower specificity scores compared to other metrics. For REC scores, U-net++ outperformed the plain U-net model by 1.2% but had the almost same score as Attention U-net by 0.1%. According to the obtained performance metrics, it can be concluded that all of the performance metrics were above 90%. The Attention U-net model produced the best overall results, despite the fact that the differences between the models were not significant (one-way ANOVA; *p* $\ge$ .999). Absolute PEs for three different U-net-based models and two different tools are given in Fig. 3c. For validation images, the absolute average PEs for U-net, U-net++, Attention U-net, ImageJ, and TScratch were yielded as 5.74%, 4.99%, 5.54%, 21.89%, and 30.52%, respectively. For test images, the absolute average PEs for U-net, U-net++, Attention U-net, ImageJ, and TScratch were yielded as 6.41%, 3.70%, 3.73%, 22.59%, and 33.88%, respectively. Figure A1 in Additional file 1 includes attention maps derived from each layer of the Attention U-net model, with the layer depth increasing. Beginning with the map from the deepest layer, where the smallest image size is achieved, the model’s focus on the edges and cell arcs is noticeable. As it proceeds to higher layers, the maps start to generalize, influenced by the low- and high-level features captured from preceding layers, and they begin to reflect the morphology present in the original image. It should be noted that, among other models, U-net++ has the lowest error values for validation and test images.

To highlight the impact of preprocessing and augmentation steps on determining the wound area, the Unet++ model was retrained using images without any pre-processing except resizing to 1024x768. This retraining was carried out under the same conditions and parameters that yielded the best results previously, but without applying any image-splitting or enhancement techniques. According to the results of the training of raw-resized images, the average DSC value was obtained as $\approx$0.942 for both the validation and test phases. This shows there is no significant difference after preprocessing and augmentation ($\ge$0.95 DSC, t-test: p=.685). However, according to the wound area determination results, the average absolute PE rates were 14.68% and 15.03% for validation and test sets, respectively. The findings highlighted a substantial average discrepancy of 9.69% and 11.33% for the validation and test sets, respectively between the two scenarios. This underscores the significant role that the preprocessing and augmentation steps play in our methodology.

**Fig. 4:**
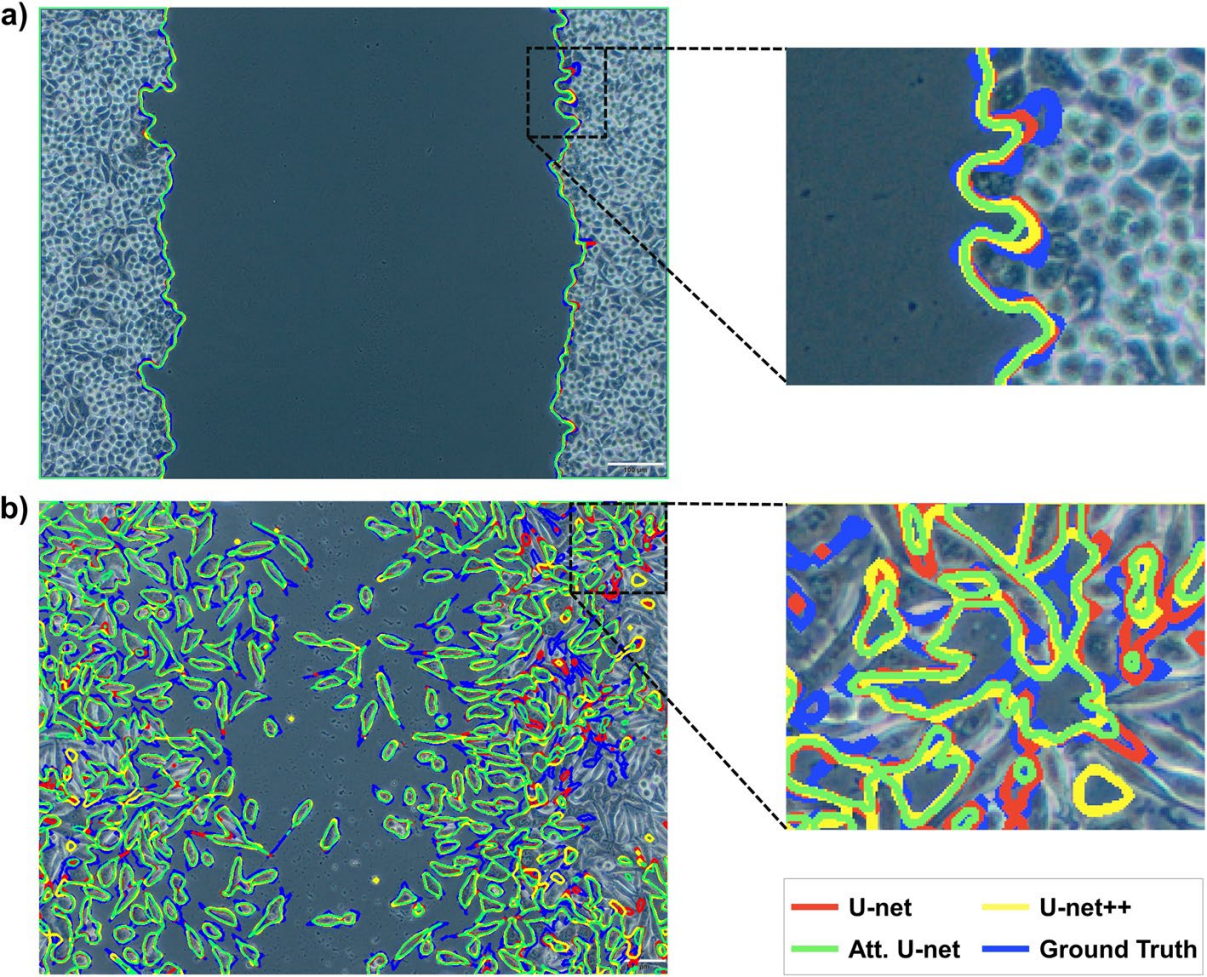
Examples of the best and worst predicted images by the developed models: **a** best-case scenario, and **b** worst-case scenario

**Fig. 5:**
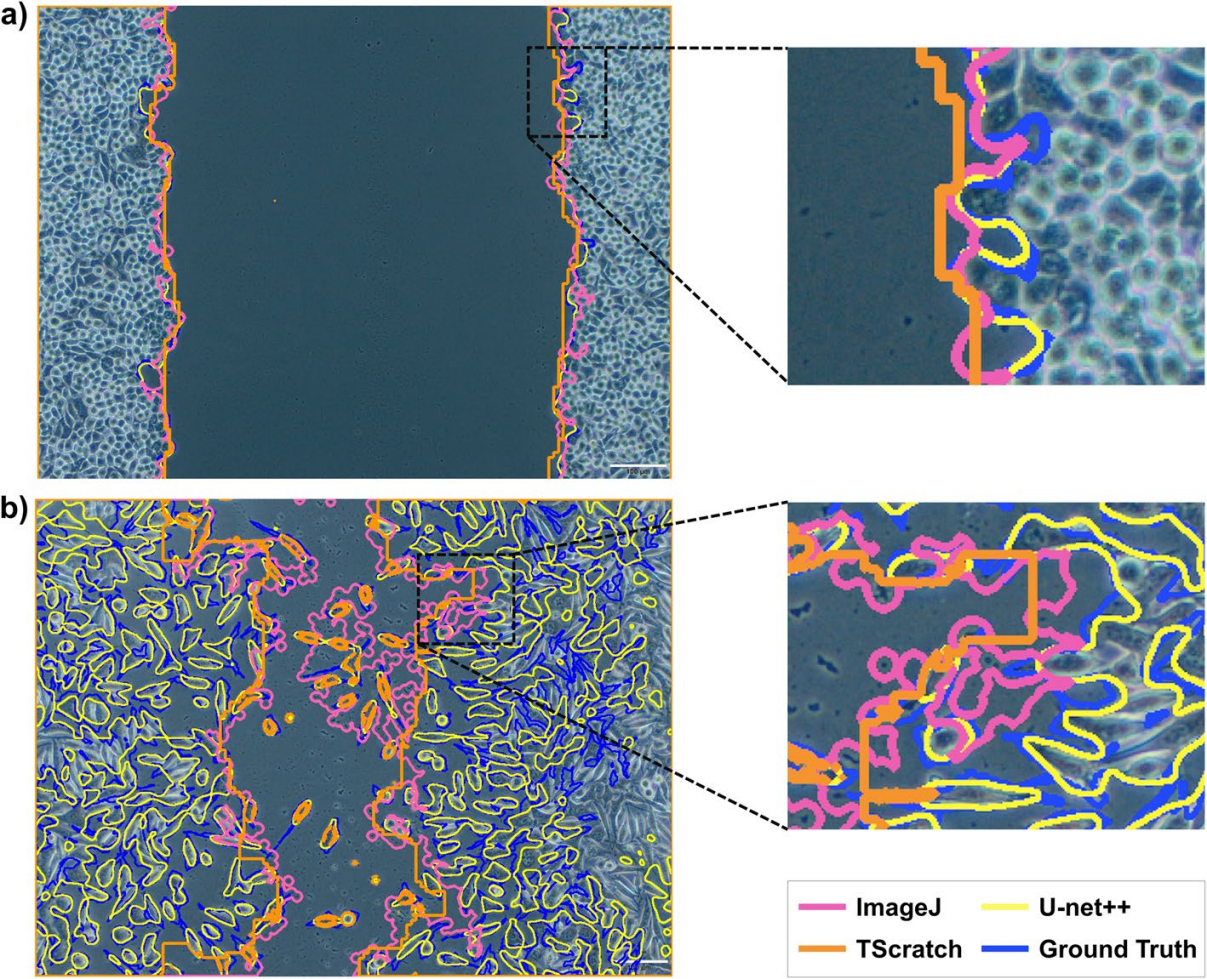
Comparison of the best and worst predicted images by U-net++ and the currently available tools for wound healing in vitro microscopy images: **a** best-case scenario, and **b** worst-case scenario

The best and worst predicted images and their comparison with the masks generated from the currently available tools are presented in Figs. 4 and 5. The best-case image (Fig. 4a) has a DSC value of 0.993 for U-net and 0.992 for U-net++ and Attention U-net models. The worst-case (Fig. 4b) has a DSC value of 0.833 for U-net and U-net++, 0.84 for Attention U-net models. More specifically, the result obtained from the U-net++ model, which produces the lowest absolute PE among the best and worst-case microscopy images in Fig. 4, was compared with ImageJ and TScratch in Fig. 5. The best-case image (Fig. 5a) has a PE of 0.09% for U-net++, 0.57% for ImageJ, and 23.62% for TScratch. The worst-case image (Fig. 5b) has a PE of 6.81% for U-net++, 60.59% for ImageJ, and 36.92% for TScratch in the calculation of wound areas. The results from each validation and test sample per model are presented in Additional file 3 and Additional file 4, along with DSC values that were calculated using the similarity between predicted areas and the actual ground truth.

**Fig. 6:**
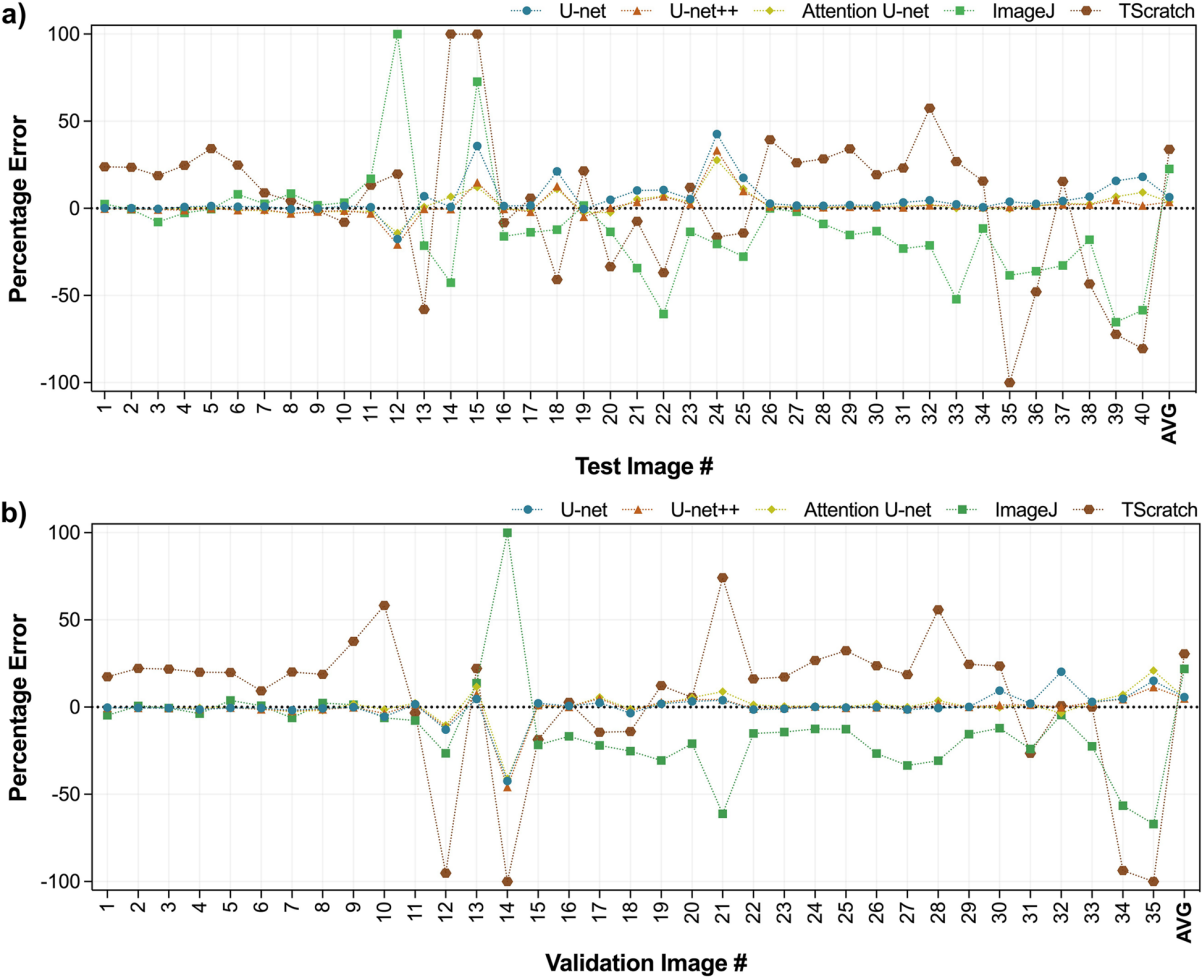
Individual sample-based PEs of predicted mask areas compared to ground truth for developed U-net models, ImageJ, and TScratch: **a** test and **b** validation samples (The absolute PE averages for U-net, U-net++, Attention U-net, ImageJ, and TScratch were 6.41%, 3.70%, 3.73%, 22.59%, and 33.88% for the test images and 5.74%, 4.99%, 5.54%, 21.89%, and 30.52% for the validation images, respectively.)

The PEs within the calculated wound areas per individual sample across all U-net models and image processing-based tools are presented in Fig. 6. The calculated areas were compared to the ground truth mask area to achieve the PE. Among test images, image #9 has the lowest PE for all models and tools compared to ground truth. The absolute PEs are as follows for U-net, U-net++, Attention U-net, ImageJ, and TScratch: 0.15%, 1.84%, 1.88%, 1.78%, and 1.40%. Among test images, image #15 has the highest PE for all models and tools compared to ground truth. The absolute PEs for U-net, U-net++, Attention U-net, ImageJ, and TScratch were achieved as 35.86%, 14.93%, 12.26%, 72.64%, and 99.94%, respectively. Regarding performance metrics, computation costs, and PE values acquired from the area calculation, it should be noted that the outcomes obtained from U-Net++ were more precise compared to not just other U-net-based models but also commonly used tools such as ImageJ and TScratch.

### External testing

**Fig. 7:**
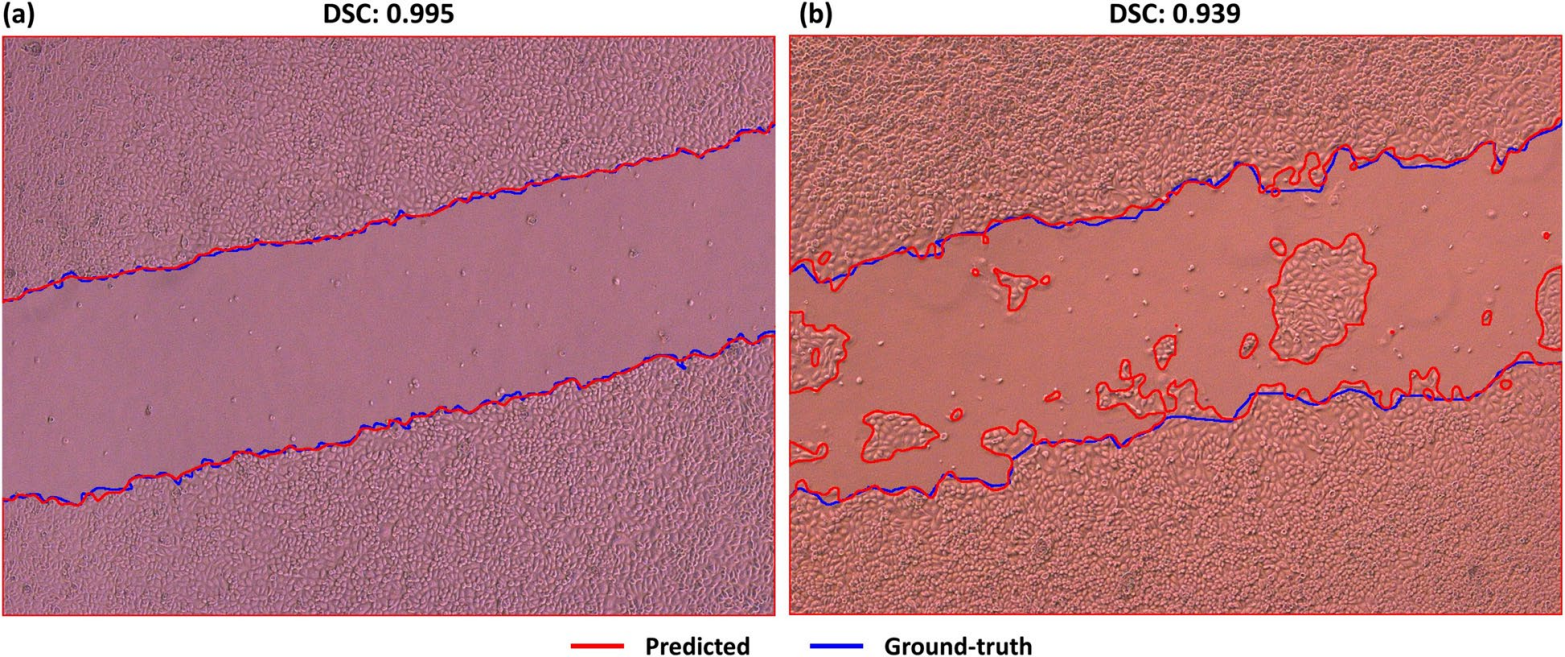
Examples of predicted contours during the external testing phase, where the ground truth is indicated by blue lines and the predicted boundaries are indicated by red lines. Examples; **a** a nearly ideal DSC and **b** effectively illustrating the developed model’s performance in predicting the migrating cells within the wound area

The effectiveness of the presented approach was assessed through external testing on a dataset published by Sinitica et al. [55]. The dataset consists of 180 images containing HuTu80 and MCF7 cell lines, along with their expert-labeled masks. In the test results, which included all 180 images, the developed model achieved an average DSC of 0.955, an accuracy of 0.948, an IoU of 0.927, a PRE of 0.966, and a REC of 0.958. Example images predicted by the developed model are shown in Fig. 7.

## Discussion

### Evaluating implications

The determination of the wound area is a crucial stage in the analysis of in vitro wound healing investigations [26]. However, toolboxes currently in use in the literature rely on assumptions about cell behavior and image processing operations that might be prone to errors, take a long time, and lack reproducibility due to their reliance on manual intervention [15]. The commonly used toolbox ImageJ has several limitations, such as the inability to separate cells, the conversion of the cells in spindle movement into circular shapes, and the fact that cells that were smaller than the radius value specified by the user were not considered cells [56]. For the white-wave model to be effective, experiment-long fixed images must be acquired, and collecting them manually exposes the application to the potential for errors [20]. Furthermore, results might differ depending on the environment, and pixel variations may be driven by cell growth [57]. Rapid analysis is possible in TScratch, but the curve coefficient must be obtained through additional processes that depend on the used parameters. Moreover, these methods are limited in their ability to accurately analyze small wound areas or adapt to different cell lines and magnifications of the microscope [58]. Consequently, the application shows inadequate robustness. They can therefore lead to inaccurate wound area calculations, resulting in incorrect or insensitive determination of therapy-of-interest [59]. Furthermore, in images where the wounds are almost closed, the determined wound area is fragmented, and in this case, a single value cannot be obtained, so there is a requirement for a combination of several calculations. Completely closed wounds have a poor success rate for discrimination [60]. The drawbacks of the already available tools make the development of alternative analysis methods attractive. To address these challenges, recent studies have revealed that DL algorithms achieve remarkably high ACC in wound healing imaging [61]. The goal of this study is to address these limitations and improve the accuracy of wound area determination. Considering these constraints, a 5-layer U-net structure based on DL and its modified variations was developed. In order to ensure the model’s independence from the cell line, magnification of the microscope, and therapy-of-interest, the dataset used for training the models was collected from 3 different cell lines, 2 different magnifications, and 2 different novel treatment methods. The highly accurate performance metrics obtained in both dataset combinations revealed that the generalizability of the proposed method was quite high. They also indicated the independence of the above-mentioned wound image generations. It should also be noted that the presented structure has an automatic detection capability of wound areas in significantly lower calculation times, which is user-independent as well. Unlikely, the manual labeling of masks for images with a 40x magnification required an average of 20 min, while images with a 100x magnification required 60 min. While this approach is not typically employed in wound healing research, it is a widely accepted practice for labeling data [62] to be used in AI-based segmentation studies. However, this boundary-marking method is quite time-consuming. This study aims to decrease this time and develop an application that minimizes the required user involvement. However, the problem here was that no research involved marking cells meticulously by accurately annotating the ROIs; instead, they utilized only parallel vertical lines to calculate the wound areas by ignoring the cell growth across various directions as discussed before [20, 63]. Despite this, while manual annotation considering each cell characteristic in wound images is quite accurate, it still has a time-consuming nature. These drawbacks result in limited data and lead to two major implications: *i)* traditional and broadband manual calculation of wound areas (based on the stretching of two horizontal lines), leading to inaccurate annotation; and *ii)* manual annotation, considering each cell’s characteristics, is accurate but time-consuming; however, it is necessary only once in the AI-based studies to accurately determine labels in the training phase.

The need for the preprocessing steps to reduce glare and reflections caused by the camera’s shooting angle and thus generate a uniform histogram distribution throughout the image demonstrated the impact on the model’s training performance. During the augmentation phase, the strategy of spatially dividing the microscopy images into four equal parts allowed for an expanded dataset size and thus enabled the maximization of detail and edge capture. Additionally, one of the key advantages of this approach lies in the fact that the masks of the images obtained through this method are also very different from each other. This allows the introduction of a greater variety of features into the training set since it might provide various versions of the edges and distinctive characteristics of the images, thereby enhancing the learning capability of our model. In contrast, traditional methods like flip and scale often result in redundant information as they typically generate transformed versions of the same image [64]. Therefore, this division approach not only increases the quantity of our training data but also significantly improves its quality by ensuring a diverse range of features for our model to learn from. The histogram equalization and augmentation steps have a significant impact on the model’s performance. The results demonstrated that the DSC value substantially increased after the preprocessing and augmentation steps.

According to training results, as the training progressed, it can be observed that the DCSs increased and stabilized towards the latter epochs, while initially showing lower and fluctuating values (Fig. 2a, e, i). The same trend is also applicable to other developed models. It could be inferred from the validation DSCs (Fig. 2c, g, k) that the Attention U-net structure exhibits greater fluctuation. This is presumed to be caused by the attention block included in each layer. Conversely, the loss values, which started off high and fluctuating, as expected, eventually decreased and stabilized near the latter epochs for both training (Fig. 2b, f, j) and validation curves (Fig. 2d, h, l). Considering the performance scores of the trained models (Fig. 3), there was no significant difference among the U-net, U-net++, and Attention U-net. The training results for the sub-datasets were found to be similar to those of the main dataset and models. However, they did differ in proportion to the size of the dataset. Despite having fewer images, it was observed that the metrics from the dataset for CAP treatment performed better compared to the dataset for LLLT. The fact that the cells are more branched because of the spindle formation and dispersed in LLLT images might be the cause. As the number of discrete cells increases, small differences occurring around the cells cause a larger deviation when viewed as a whole image. Furthermore, the high-performance outcomes observed in CAP treatment images provide evidence that the model can produce highly accurate predictions regardless of the cell line and magnification of the microscope. However, it should also be noted that the model performances were relatively accurate ($>0.95$ DSC) in all three datasets. This implication revealed the generalizability of the proposed methodology regardless of the various dataset combinations and therapy-of-interest. For the LLLT and CAP treatment datasets, corresponding DSC, ACC, IoU, PRE, REC, SPE, and ROC-AUC scores were close to each other for the three developed models (Table 3). However, in the test of the LLLT data, the IoU metric demonstrated relatively lower results. Similarly, the SPE metric showed lower results for the CAP data. This could be because datasets might have unique characteristics that make it more challenging for certain metrics to perform well. For example, IoU might struggle with overlapping or closely situated objects, which could be more common in the LLLT data. Similarly, the SPE metric might be more sensitive to the true negative rate, which could be more prevalent in the CAP data. Additionally, considering the time required to establish a ground truth mask, it can be observed that all three models developed significantly reduce the computational cost. In particular, the U-net++ model produces outcomes faster than those of other models. This is attributed to the process of dense skip blocks, which involves a smaller number of parameters in the model. It may also be noted that Unet++ underperformed in certain metrics, such as SPE and PRE.

Additionally, the DSC averages were separately computed for the 40x and 100x images, and then an unpaired nonparametric two-tailed t-test based on the Mann-Whitney [65] test was conducted to determine if there was a statistical difference between the two groups. The results revealed a difference in the test data with a *p*-value less than 0.0001. Statistical significance was also observed in the validation data, with a *p*-value of 0.026. By filtering the results, the 40x magnification yielded higher average DSCs of 0.992 in the test data and 0.994 in the validation data. Also, the model performed competitively, with an average DCS exceeding 0.95 at 100x magnification. This was attributed to the fact that as the image magnification increases, more details are captured and the model predicts a larger number of features, resulting in slightly decreasing performance. Given the relatively accurate performance (DSC of 0.95) achieved at larger magnifications (100x), the results were in line with the expectation that the model would be even more successful at predicting images at smaller magnifications (DSC of 0.992 at 40x).

The best and worst scenario results (Fig. 4) demonstrate that all models were consistent with the ground truth. Therefore, it can be concluded that the employed models have shown promising performances. The disparities here can be explained by slight boundary deviations brought on by the cells’ fragmented nature, resulting in a greater variance when the whole image is considered. Comparing the models, the Attention U-net captures the details closer to the ground truth than other models because of the AGs in its structure. Although boundaries were similar to the ground truth, hence, the DSC values were very close for all models, when the PE values for wound area calculation were compared (Fig. 6), U-net++ significantly outperformed the other models. Furthermore, even if the outcomes provided by the developed models are remarkably similar to one another, it can be claimed that U-net++ was superior to other models considering both the metrics, computational cost, and average absolute PE outcomes.

The best and worst scenario results (Fig. 5) revealed huge deviations in ImageJ and TScratch tools. Compared to the ground truth, it has been observed that the mask produced by Tscratch produces results that neglect the circular structure of the cells. The scenario was the opposite for ImageJ. In ImageJ, cells were estimated more circularly, resulting in decreased precision, especially in cells that are in the spindle movement stage. The performance differences become much clearer when comparing the ImageJ and TScratch tools with ground truth and U-net++. U-net++ has almost the same borderlines as ground truth. This once again emphasizes the superiority of the developed model in calculating the wound area in a specific, sensitive, and accurate way compared to the currently available tools. In fact, when the test and validation images are analyzed one by one and the PEs are obtained (Fig. 6), the individual variability in each image for the ImageJ and TScracth tools stands out. In addition to the shortcomings of these tools, their computational capability for different images varies greatly. However, when the developed U-net-based models are analyzed, it can be concluded that these models gave a result similar to the ground truth for each image, and there is no individual variability. Based on these, the developed models have the potential to be more accurate than the current tools and methodologies employed to calculate the wound area.

### Comparison with related studies

In biomedical imaging, DL techniques have been utilized for various operations such as segmentation, classification, and detection [66]. CNNs, a sub-branch of DL, have been utilized for semantic segmentation, where each pixel is classified with a specific label. While ML applications have their own set of challenges, such as the necessity for handcrafted feature extraction and potential performance degradation in the face of high-dimensional data compared to DL [37, 38], research on the schematic analysis of wound healing microscopy images using ML continues to be reported. In the field of ML, MultiCellSeg is a tool that utilizes the statistical learning of Support Vector Machines (SVMs) to segment images. During the training phase of the study, basic image attributes are used to classify the images labeled as cellular regions and backgrounds into regional patches [67]. This process is called patch classification. The model analyzes the patches in the region by grouping them independently, taking into account the image-texture information, and using the Graphic-segment-based segmentation application to determine the areas with and without cells. Furthermore, Glaß et al. developed a method for area segmentation based on image classification that evaluates the wound border and area using level-set techniques before excluding non-scratch images with SVMs [68]. They employed an entropy-based energy function and extended non-partial differential equation level sets to maintain the topology in the level set procedures. The bottom row median REC value was reported as 0.88 on average and the PRE value as 0.87, whereas the top row median REC value was 0.80 and the PRE value was 0.93, indicating the quantitative success of the model. They claimed that the technique they developed could be implemented as an ImageJ plugin, required minimal input parameters, and was suitable for experimental evaluations.

On the DL side, Oldenburg et al. developed a platform for living cell research that can conduct cell- and population-scale analyses using MATLAB-based DL techniques [25]. They introduced a system that can analyze cell mobility at both the cell and population scales by training a 3-layer U-net structure using a semi-automatic labeling method. In the mentioned study, they performed the numeric evaluation of the leading edge with a second DL method called edge protrusion. They reported their success in cell detection and segmentation with an IoU score of 0.8214±0.038. Ayanzadeh et al. developed a new architecture by utilizing an alternative feature extractor in the U-net encoder and replacing the plain blocks in the decoder with residual blocks [30]. These modifications were based on the shortcomings of the naive U-net model and aimed to improve its performance. For segmentation, U-Net and a pre-trained ResNet-18 encoder were used. A novel skip connection was proposed to reduce the semantic gap between the encoder and the decoder, and it was determined that this skip connection improved the model accuracy across both datasets. In the DSB2018 and MDA-MB-231 datasets, the suggested segmentation method produced Jaccard Index values of 85.0% and 89.2%, respectively. Another AI-based approach is the DeepScratch application, developed by Javer et al., which utilizes a U-net structure to identify nuclear or membrane images from heterogeneous image data [69]. The authors used dot marking to annotate cells in HDLECs scratch assay images at 0 and 24 hours and subsequently trained the model. To segment wounds, cell-free regions were considered as wounds. The coordinates of the cells were transformed into a mask with pixel-by-pixel annotations, and the cell density was determined by applying a uniform 13x13 pixel kernel to the masks. A morphological opening with a 35x35-pixel kernel was applied, and any black pixels were classified as corresponding to the wound area to construct a segmentation mask. They identified all connected components in the wound mask, and the wound was determined as the object with the largest area. The performance of this developed method is reported as 91.7% PRE, 92.1% REC, and 92.5% F score for mixed sets, and 95.4% PRE, 96.2% REC, and 95.8% F score for mixed sets+nuclei.

Sinitca et al. developed a segmentation application for the semi-automatic segmentation of images based on their patchiness using local edge density estimates. To segment and quantify the image, they initially performed various image processing operations, such as edge detection, local edge density, and thresholding. The parameters in the process were optimized using pixel densities and regression analyses of the image. A modified U-net model (U-netR) was then trained with both masks created by experts and masks generated as a result of automatically adjusted parameters with the developed application interface. The success of the results obtained has been reported with a median ACC of 95-99%. The research involved the use of several cell lines and microscope magnifications. Although this has an important value in terms of the generalizability of the application, it requires parameters directly controlled by the end user for segmentation [55]. To assess the generalizability of our model further, we tested 180 wound-healing images published by Sinitca et al. This evaluation allowed us to observe the models’ performance under different scenarios and identify potential areas for improvement. It is important to note that all images in this dataset were captured at a 40x microscope magnification. The obtained results of our model (>0.95 DSC) utilizing this external dataset highlight its robustness and adaptability. Despite significant differences in the dataset, including microscope magnification, different lighting conditions, and the orientation of the scratch assay, our model achieved competitive results. This underscores the model’s ability to generalize across diverse conditions, which is a crucial attribute for practical applications. Additionally, the absence of user intervention or parameter tuning in our methodology simplifies the process and enhances reproducibility.

As explained above, there is no standard approach to the segmentation of wound healing microscopy images yet. The previous research focused on high segmentation performance by using a different number of input data, cell lines, or DL architectures. This makes the fair comparison of recent methods challenging, but the comparison of the capability of generalizability is still important. The summarization of the above-mentioned studies and current work is presented in Table 4. It can be observed that the majority of the metrics yielded competitive outcomes. It is important to note that the methodologies employed in the referenced studies were based on their specific, custom datasets, which are not publicly available and involve different experimental strategies for the scratch assay process. Despite these differences, we strive to present a comprehensive framework that enables us to demonstrate the performance of similar studies in the process of wound healing segmentation. The results we obtained indicate that the methodology we presented is promising when compared to existing methods. This underscores the superiority of DL-based applications, consistent with the research discussed in previous sections. This demonstrated that DL techniques can be employed to analyze wound healing images and provide highly accurate segmentation. Moreover, our approach demonstrated high accuracy and robustness in their segmentation performance regardless of the user across a variety of conditions, including therapy-of-interest, cell lines, and magnification of the microscope, while most of the others had not considered these important experimental-related variables. It is also important to note that these methods depend on particular conditions and require input for key parameters. Therefore, it is still crucial to improve these techniques by establishing standardized and generalized analysis methods that are independent of the particular images being examined or the application-specific alterations. This would make the methods more widely applicable in the field of wound healing research and increase the consistency and reliability of the obtained results. Moreover, the development of a standard methodology would also make it easier to compare the outcomes across different studies and experiments, thus facilitating the advancement of our understanding of the wound healing process and the efficacy of the therapy-of-interest. Last but not least, it should be noted that scratch wound healing assays are not only used for simulating actual wound healing but also serve as a robust tool for evaluating cell motility and migration. These cellular behaviors are integral to numerous biological processes, thereby extending the relevance of our method beyond wound healing.

**Table 4:** Comparison of current work with state-of-the-art studies in literature. The given metrics represent the most effective results derived from corresponding research. In the metrics that were reported as subsets in studies, the median average values of corresponding metrics are included in this table. Also, in cases where studies report only validation scores, those scores were included instead of test results. $\mathcal {O}$ represents the computational complexity value. Computational complexity varies according to model layers, parameters, and input dimensionality. Here *L* is the number of layers in the U-Net model, $L_{r}$ is the number of layers in the ResNet encoder, $L_{u}$ is the number of layers in the U-Net decoder, *n* is the input size, *k* is the filter size, *d* is the number of input channels, and *f* is the number of filters. $L_{a}$ is the number of additional layers due to attention mechanisms; $L_{n}$ is the number of additional nested layers in U-Net++; and $L_{t}$ is the number of transformer layers (refer [70] further investigation on the computational complexity of CNNs). Note that the $\mathcal {O}$ notation is not fixed and may vary based on the input size and the specific details of the implementation [71]. The representation given here is intended to serve as a general guide to understanding the complexity of the model

Study	Cell line	Data type	Sample size	Architecture	DSC	ACC	IoU	PRE	REC	SPE	Network complexity
Zaritsky et al. [67]	DA3, MDCK	Wound healing assay	126	SVMs	N/A	0.945	N/A	N/A	N/A	N/A	$\mathcal {O}(n^2)$
Glass et al. [68]	U2OS, 8505C	Wound healing assay	107, 60	SVMs	N/A	N/A	N/A	0.900	0.840	N/A	$\mathcal {O}(n^2)$
Oldenburg et al. [25]	HCAEC	Wound healing assay	280	U-net	0.918	N/A	0.821	N/A	N/A	N/A	$\mathcal {O}(L \cdot n^2 \cdot k^2 \cdot d \cdot f)$
Ayanzadeh et al. [30]	MDA-MB-231,DSB2018	Individual cell segmentation	600, 670	U-net, ResNet-18	0.943	N/A	0.892	0.958	0.928	N/A	$\mathcal {O}((18 + L_u) \cdot n^2 \cdot k^2 \cdot d \cdot f)$
Javer et al. [69]	HDLECs	Wound healing assay & Individual cell segmentation	90	U-net, ResNet	0.958	N/A	N/A	0.954	0.962	N/A	$\mathcal {O}((L_r + L_u) \cdot n^2 \cdot k^2 \cdot d \cdot f)$
Sinitca et al. [55]	HuTu 80, MCF7	Wound healing assay	180	U-netR	0.977	0.970	0.955	N/A	N/A	N/A	$\mathcal {O}(L_u \cdot n^3 \cdot k^3 \cdot d \cdot f) + O(L_t \cdot n^6)$
This Work	L929, HS2, SCC	Wound healing assay	233, 133, 34	U-net	0.959	0.961	0.926	0.975	0.945	0.917	$\mathcal {O}(L \cdot n^2 \cdot k^2 \cdot d \cdot f)$
				U-net++	0.958	0.962	0.928	0.965	0.957	0.905	$\mathcal {O}( (L + L_n) \cdot n^2 \cdot k^2 \cdot d \cdot f )$
				Attention U-net	0.960	0.964	0.932	0.968	0.958	0.914	$\mathcal {O}( (L + L_a) \cdot n^2 \cdot k^2 \cdot d \cdot f)$

### Limitations

Besides the promising results, several limitations were encountered that could have potentially influenced the results. Firstly, the limited amount of data available for this study might have restricted the model’s ability to learn more complex patterns, thereby affecting its generalizability and robustness. This is a common issue in wound healing studies due to public dataset limitations and non-standard labeling, often leading to the use of custom datasets. Consequently, the dataset used in this study was retrospectively collected from previous studies within the laboratories. To the best of our knowledge, the dataset, which comprises 400 different wound healing microscopy images, is one of the largest used in this context. To mitigate the impact of data limitations on model performance and to enhance the information obtained from the edges, we augmented the training data using a spatial partitioning process commonly used in handling high-dimensional images. This process caused an additional preprocessing step, which could be another drawback. The necessity for an additional preprocessing step added a layer of complexity to the data preparation process. This might pose a challenge for the scaling of the study. The other limitation may be the presence of unusual peak outliers in the validation curves. While these peaks could be present due to various reasons, they were not excluded to ensure a fair comparison of the performances of the developed models. The peaks in the validation scores may cause a delay in the computational time. However, it is important to note that we need to maintain a consistent number of epochs across all runs to ensure a fair comparison in terms of computational time. All models are trained for 50 epochs to avoid discrepancies that could arise from varying training durations or the use of early stopping mechanisms. Lastly, especially the performance of the external test samples, depends on the provided masks. This causes the DSC calculation to be based on the provided masks. In cases where the cell boundaries were not annotated well, the DSC scores will decrease even if the model predicts wound areas better since the similarity comparison is based on the ground truth (which was not annotated meticulously).

## Conclusion

In this study, a U-net-based DL model was developed to provide a standardized approach for the calculation of wound area from in vitro microscopy images. The model was trained by utilizing in vitro wound healing images of three different cell lines from LLLT and CAP treatment investigations. The performance of the model was evaluated using various metrics during a robust and comprehensive testing phase, and a success rate of over 90% was achieved in all metrics. This process was repeated with slightly different variations of the U-net model. The DSC values were obtained around 0.958 for all 5-layer U-net, U-net++, and Attention U-net models, while U-net++ had the best wound area calculation performance. The deviation of the wound area calculation from the ground truth was also substantially lower for Unet++ (3.70%) than ImageJ (22.59%) and TScratch (33.88%). Although the sample size does not allow for extensive generalizability evaluation, the performance of the proposed method on the external sets was consistent, as evidenced by the low variability across three different datasets. Overall, the developed method has produced satisfactory outcomes when compared to the most recent studies in the literature and enables fast and more standardized analysis.

### Supplementary information

Additional file 1. A brief outline of CAP and LLLT treatments, and the attention maps derived from each layer of the Attention U-net model.

Additional file 2. Detailed model summaries for U-net, U-net++, and Attention U-net.

Additional file 3. Validation phase results (contour drawings) for U-net, U-net++, and Attention U-net models as separated by individual samples.

Additional file 4. Test phase results (contour drawings) for U-net, U-net++, and Attention U-net models as separated by individual samples.

## Data Availability

The data that support the findings of this study are available on request from the corresponding author.

## Abbreviations

ACC: Accuracy
AG: Attention Gate
AI: Artificial Intelligence
AIM: Automatic Invasiveness Measure
CAP: Cold Atmospheric Plasma
CLAHE: Contrast Limited Adaptive Histogram Equalization
CNNs: Convolutional Neural Networks
DL: Deep Learning
DSC: Dice Similarity Coefficient
FN: False Negative
FP: False Positive
GANs: Generative Adversarial Networks
HS2: Human keratinocyte
IKCU: Izmir Katip Celebi University
IoU: Intersection over Union
LLLT: Low-level Laser Therapy
ML: Machine Learning
MPS: Microphysiological Systems
PE: Percentage Error
PRE: Precision
REC: Recall
ROC-AUC: Receiver Operating Characteristics-Area Under The Curve
ROI: Region of Interest
SCC: Squamous Cell Carcinoma
SPE: Specificity
SVMs: Support Vector Machines
TN: True Negative
TP: True Positive
WA: Wound Area
