# Supplementary material for: An automated in vitro wound healing microscopy image analysis approach utilizing U-net-based deep learning methodology
Source: BMC Med Imaging. 2024 Jun 25;24:158. doi: 10.1186/s12880-024-01332-2 (PMC11197287; doi:10.1186/s12880-024-01332-2)

Additional File 1. – Brief explanations on the low-level laser therapy (LLLT) and cold atmospheric plasma (CAP) treatment.

Low-Level Laser Therapy (LLLT): LLLT is a non-invasive method that stimulates cellular activity and promote tissue restoration by using low-intensity lasers or light-emitting diodes (LEDs). The therapy includes subjecting tissues to particular light wavelengths, usually in the red or near-infrared spectrum, which penetrate the skin and interact with chromophores within the cells (Farivar et al., 2014). This interaction leads to a cascade of biological processes, including increased ATP production, modulation of reactive oxygen species (ROS), and release of growth factors, ultimately promotes cellular proliferation and migration. LLLT is widely recognized for its capability to accelerate the healing process without causing thermal damage to tissues, and it is frequently utilized in clinical settings for pain management, inflammation reduction, and wound healing (Al-Watban et al., 2007).

Cold Atmospheric Plasma (CAP) Treatment: CAP treatment involves the application of ionized gas at room temperature to biological tissues. CAP is produced by applying a high-voltage electric field to a gas between two electrodes, creating a mixture of reactive species, including oxygen and nitrogen species (RONS), ions, electrons, and neutral particles (Bárdos and Baránková, 2010). This ionized gas can be applied directly to wounds or other tissues, promoting wound healing through different cellular mechanisms. CAP treatment has been shown to enhance cellular proliferation and migration, modulate inflammation, and exert antimicrobial effects by inactivating pathogens. The unique combination of reactive species produced by CAP interacts with cellular and molecular components, leading to improved and accelerated wound healing outcomes (Arndt et al., 2013). Due to its diverse biological effects, CAP is gaining attention as a novel and efficacious therapy approach for infections, chronic wounds, and other medical disorders (Izadjoo et al., 2018).

Additional References

Al-Watban, F. A., Zhang, X. Y., & Andres, B. L. (2007). Low-level laser therapy enhances wound healing in diabetic rats: a comparison of different lasers. *Photomedicine and laser surgery*, 25(2), 72-77.

Arndt S, Unger P, Wacker E, Shimizu T, Heinlin J, et al. (2013) Cold Atmospheric Plasma (CAP) Changes Gene Expression of Key Molecules of the Wound Healing Machinery and Improves Wound Healing In Vitro and In Vivo. *PLOS ONE,* 8(11), e79325

Bárdos, L., & Baránková, H. (2010). Cold atmospheric plasma: Sources, processes, and applications. *Thin solid films*, 518(23), 6705-6713.

Farivar, S., Malekshahabi, T., & Shiari, R. (2014). Biological effects of low level laser therapy. *Journal of lasers in medical sciences*, 5(2), 58–62.

Izadjoo, M., Zack, S., Kim, H., & Skiba, J. (2018). Medical applications of cold atmospheric plasma: State of the science. *Journal of Wound Care*, 27(Sup9), S4-S10.

Additional File 1. – Figure A1: Attention maps derived from each layer of the Attention U-net model. (a) represents the outermost layer with a resolution of 384x512; (b) represents the 3rd layer with a resolution of 192x256; (c) represents the 2nd layer with a resolution of 96x128, and (d) represents the deepest layer with a resolution of 48x64.

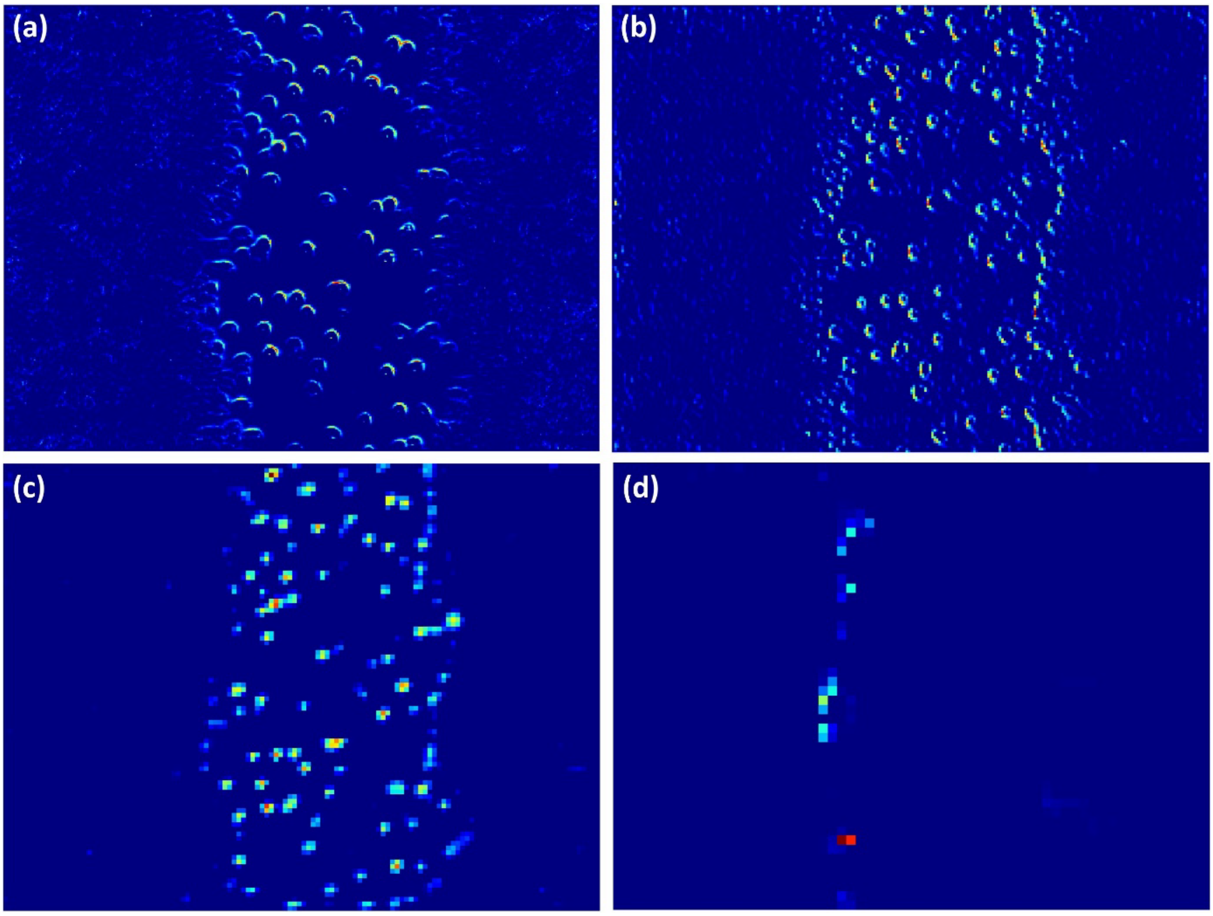

Supplement: Supplementary file 1 — Additional file 1. A brief outline of CAP and LLLT treatments, and the attention maps derived from each layer of the Attention U-net model. [file 12880_2024_1332_MOESM1_ESM.pdf]
