# Supplementary figures and images for: An automated in vitro wound healing microscopy image analysis approach utilizing U-net-based deep learning methodology
Source: BMC Med Imaging. 2024 Jun 25;24:158. doi: 10.1186/s12880-024-01332-2 (PMC11197287; doi:10.1186/s12880-024-01332-2)

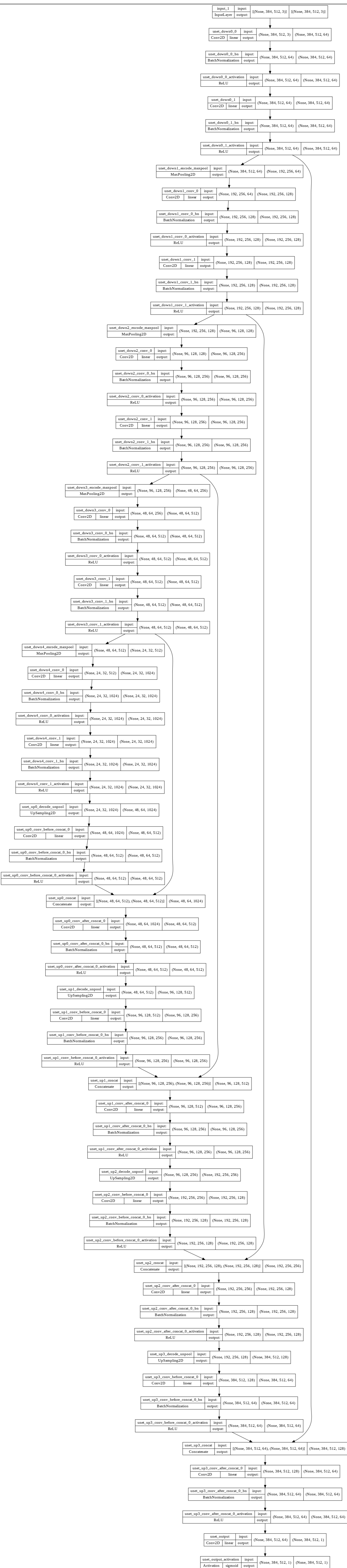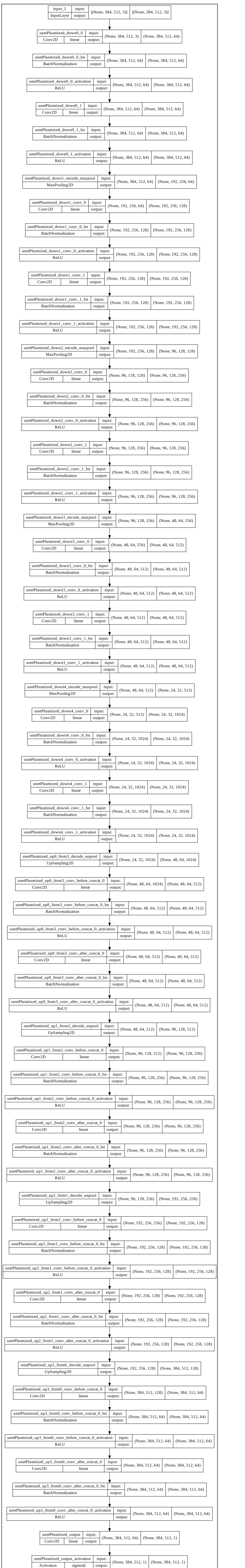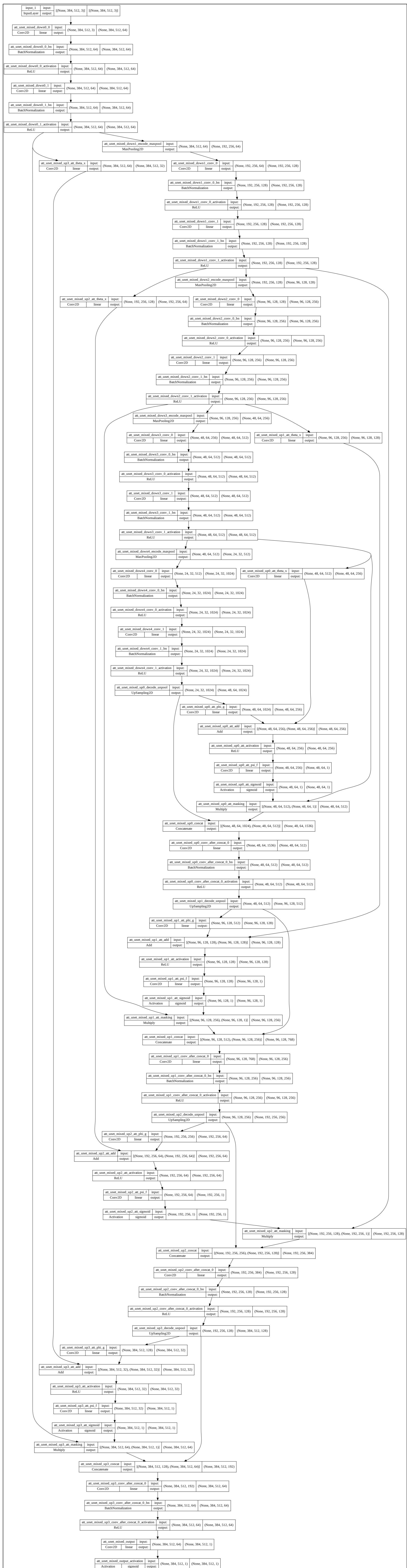

Supplement: Supplementary file 2 — Additional file 2. Detailed model summaries for U-net, U-net++, and Attention U-net. [file 12880_2024_1332_MOESM2_ESM.pdf]
