# Supplementary material for: An automated in vitro wound healing microscopy image analysis approach utilizing U-net-based deep learning methodology
Source: BMC Med Imaging. 2024 Jun 25;24:158. doi: 10.1186/s12880-024-01332-2 (PMC11197287; doi:10.1186/s12880-024-01332-2)

**Additional File 4 – Figure A1:** Test phase results for **U-net** model. Each test image sample showed as in its original form, and the predicted and ground truth labels contours were drawn on the image to observe the edge prediction discrimination. Contour lines were denoted as Red (-) for Predicted and Blue (-) for Ground-Truth (DSC: Dice Similarity Coefficient).

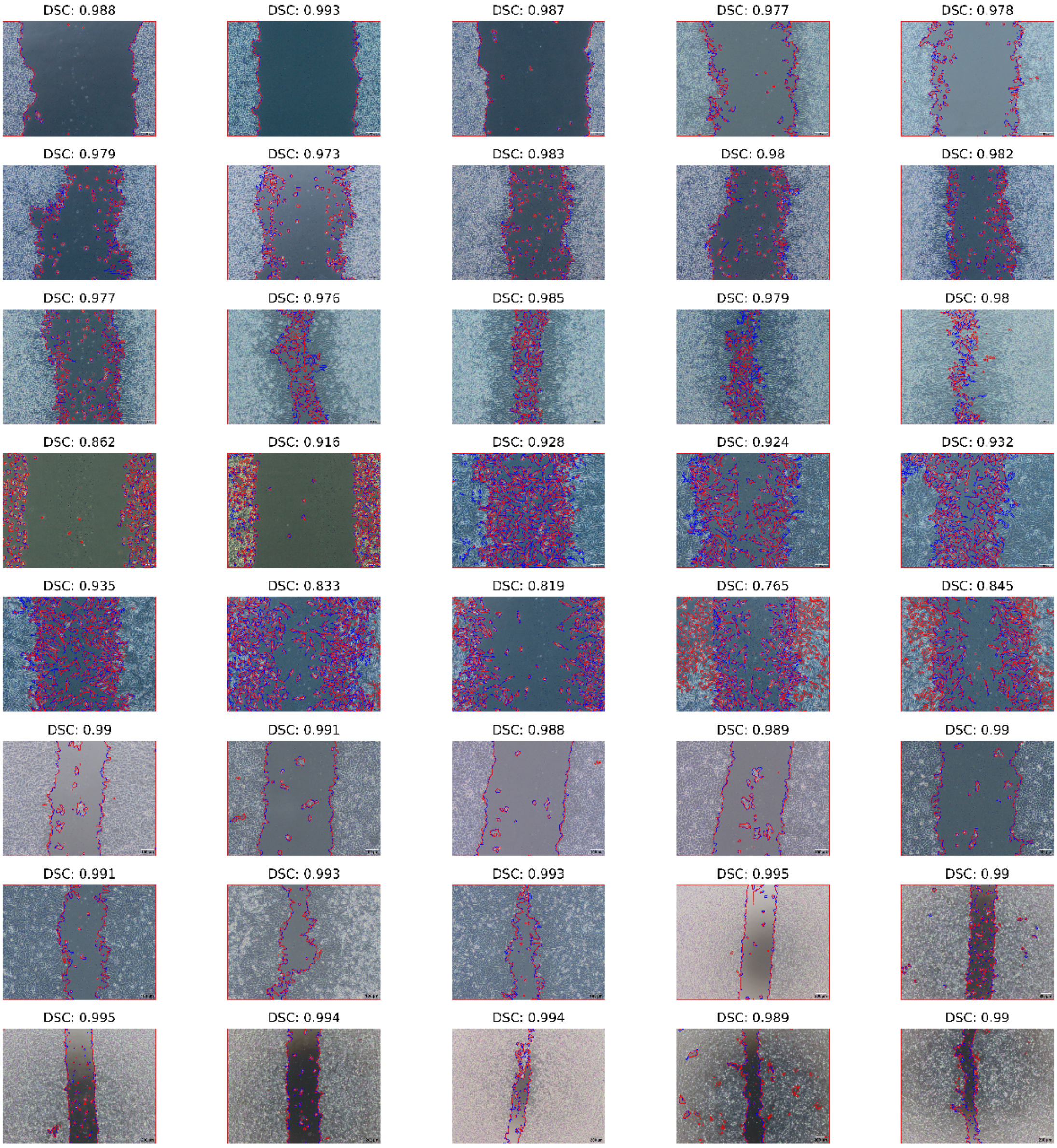

**Additional File 4. – Figure A2:** Test phase results for **U-net++** model. Each test image sample showed as in its original form, and the predicted and ground truth labels contours were drawn on the image to observe the edge prediction discrimination. Contour lines were denoted as Red (-) for Predicted and Blue (-) for Ground-Truth (DSC: Dice Similarity Coefficient).

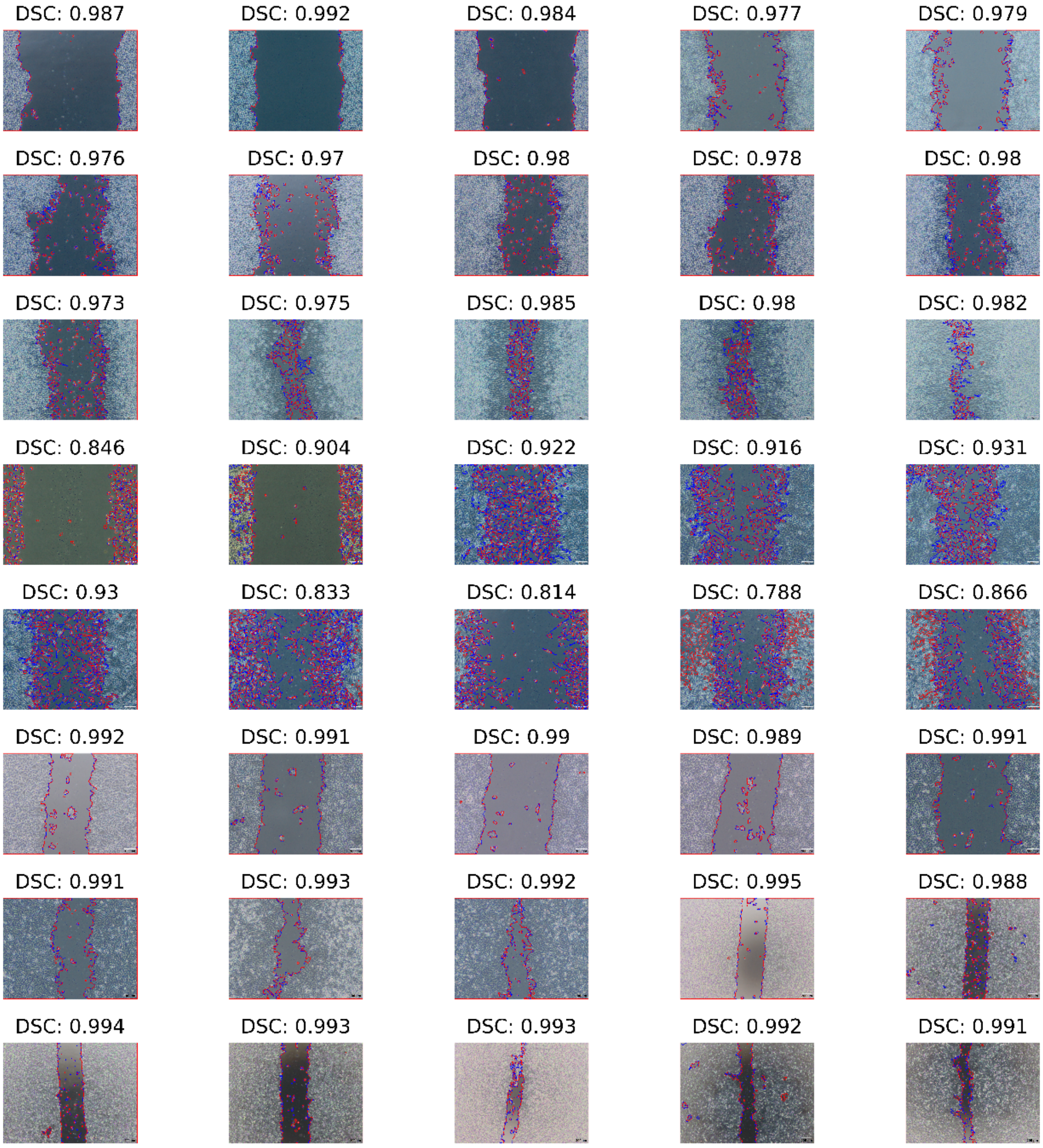

**Additional File 4. – Figure A3:** Test phase results for **Attention U-net** model. Each test image sample showed as in its original form, and the predicted and ground truth labels contours were drawn on the image to observe the edge prediction discrimination. Contour lines were denoted as Red (-) for Predicted and Blue (-) for Ground-Truth (DSC: Dice Similarity Coefficient).

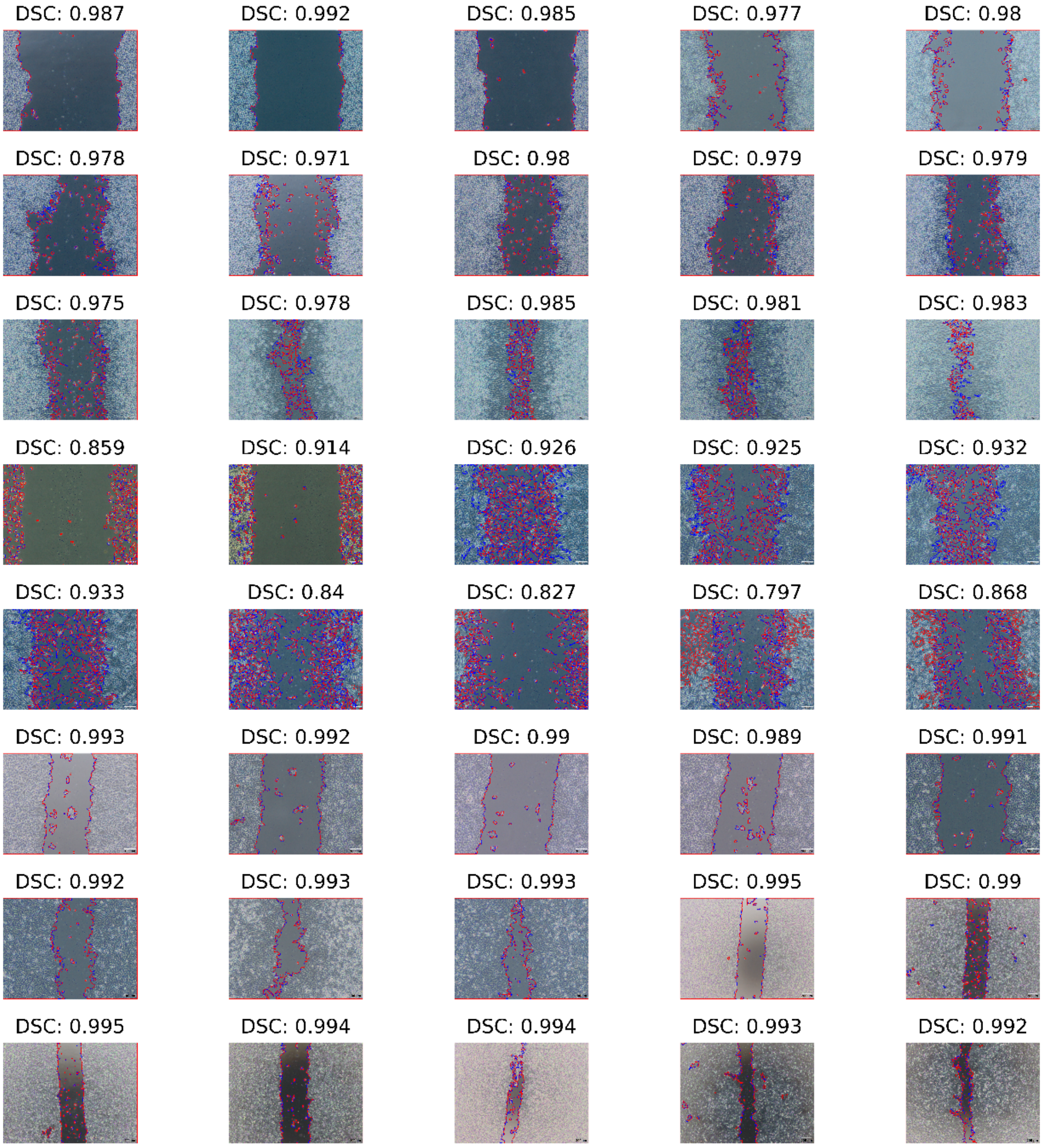

Supplement: Supplementary file 4 — Additional file 4. Test phase results (contour drawings) for U-net, U-net++, and Attention U-net models as separated by individual samples. [file 12880_2024_1332_MOESM4_ESM.pdf]
